# Metal-catalyzed coupling/carbonylative cyclizations for accessing dibenzodiazepinones: an expedient route to clozapine and other drugs

**DOI:** 10.3762/bjoc.20.19

**Published:** 2024-01-31

**Authors:** Amina Moutayakine, Anthony J Burke

**Affiliations:** 1 Instituto Universitario de Bio-Orgánica “Antonio González” (IUBO-AG), University of la Laguna, 38206 San Cristóbal de La Laguna, Santa Cruz de Tenerife, Spain; 2 LAQV-REQUIMTE, University of Évora, Rua Romão Ramalho, 59, 7000 Évora, Portugal; 3 University of Évora, Department of Chemistry Rua Romão Ramalho, 59, 7000 Évora, Portugal; 4 Faculty of Pharmacy, University of Coimbra, Pólo das Ciências da Saúde, Azinhaga de Santa Coimbra, 3000-548 Coimbra, Portugal

**Keywords:** Buchwald–Hartwig, carbonylative cyclization, Chan–Lam, nitrogen heterocycle, one-pot

## Abstract

A sequential strategy to access 10,11-dihydro-5*H*-dibenzo[*b*,*e*][1,4]diazepinones (DBDAPs) is disclosed in this article through a palladium and copper-catalyzed amination (Buchwald–Hartwig (B–H) or Chan–Lam (C–L)) followed by a palladium-catalyzed intramolecular aminocarbonylation with Mo(CO)_6_ as CO surrogate (to avoid toxic CO handling) of readily available *o*-phenylenediamines and either 1,2-dibromobenzene or 2-bromophenylboronic acid. The 10,11-dihydro-5*H*-dibenzo[*b*,*e*][1,4]diazepinone could be synthezised in good yield using a sequential catalytic procedure, using both C–L and B–H approaches. Gratifingly, the use of the C–L reaction was more impressive, and afforded the dibenzodiazepinones in good yields (up to 45%; 2 steps) and much milder conditions using copper as the catalyst. The synthetic utility of this novel strategy was showcased by demonstrating a formal synthesis for the antipsychotic drug clozapine and to an anticancer triazole–DBDAP hybrid.

## Introduction

**Figure 1:**
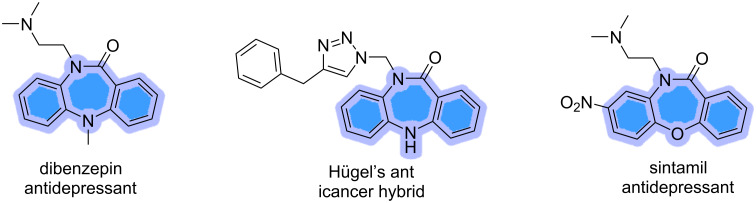
Biologically active dibenzodiazepinones.

Dibenzodiazepine units are without doubt highly privileged structures, endowed with numerous medicinally relevant properties, and notably include anti-anxiolytic and antidepressant activities. These scaffolds have received much interest from the medicinal chemist community, which led to the development of several antidepressant agents such as dibenzepin, sintamil, as well as the well-known medication, clozapine, an FDA-approved atypical antipsychotic drug, that has been adopted as a treatment of schizophrenia and schizoaffective disorders (Figure 1) [1–2]. Dibenzodiazepinones were also found to exhibit significant anticancer properties [3], as they were found to effectively inhibit tumor invasion in vitro [4], and induce apoptosis among several cancer cell lines [5]. Additionally, several dibenzodiazepinone-based structures were proven to act as p21-activated kinase (PAK) inhibitors [6], and Chk1 inhibitors [7]. The abovementioned pharmaceutical properties of the dibenzodiazepinone class have driven the development of novel synthetic strategies leading to these scaffolds in a step-economical and greener manner. Our previous review in 2018 focused on a variety of routes to these compounds [8].

The well-known Buchwald–Hartwig (B–H) and Chan–Lam (C–L) reactions have proven to be highly useful procedures that allow the step-economical synthesis of diverse biologically relevant heterocycles through C–N bond formation [9]. These approaches resulted in shortening the synthetic routes that were widely employed to access these heterocyclic scaffolds. Over the last decades, the Chan–Lam coupling reaction has drawn great attention among the synthetic chemistry community which contributed to the development of various synthetic routes to relevant heterocycles in high efficiency [10]. The Chan–Lam coupling is considered a greener alternative to traditional C–N coupling reactions, as it can be carried out under mild reaction conditions (room temperature and short reaction times, etc.), plus it does not require expensive metals like Pd, being carried out with Cu.

The aminocarbonylation reaction which was introduced by Schoenberg and Heck in 1974 is an efficient catalytic route to carboxamides [11]. It was a major step forward and has been amply applied in a number of carbonylation reactions over the years [12].

**Scheme 1:**
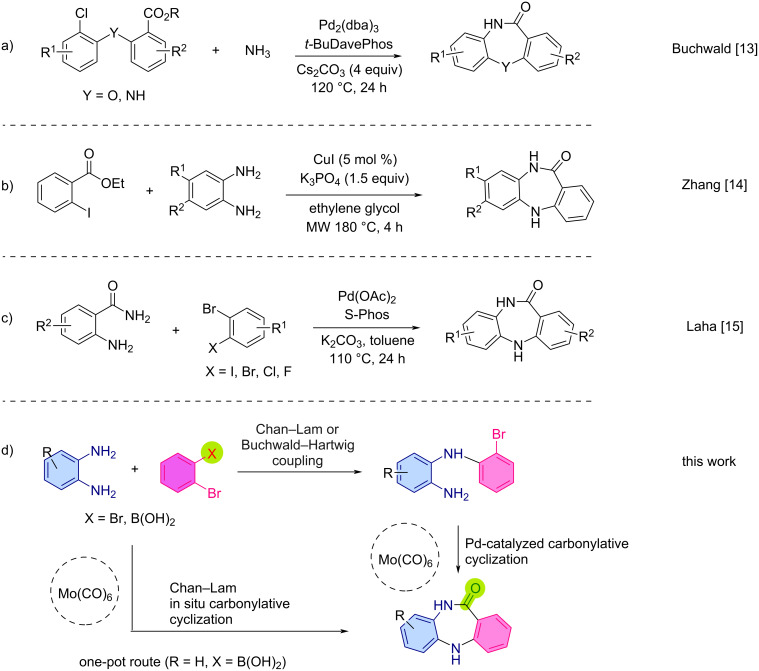
Different synthetic routes to DBDAPs (a–c), including our novel approach (d).

In 2011, Buchwald and Tsvelikhovsky introduced an efficient synthetic strategy to construct diverse dibenzodiazepinones through a sequential methodology consisting of a B–H coupling between *o*-carbonylanilines and 1,2-dihaloarene derivatives providing access to key precursors that undergo a tandem amination–intramolecular cyclization via a cross-coupling reaction with NH_3_ [13]. The reaction was undertaken in the presence of a catalytic amount of a palladium catalyst and afforded a library of dibenzodiazepinones in good to excellent yields (Scheme 1a).

In 2013, Zhang et al. developed a synthetic route leading to structurally diverse dibenzodiazepinones via a copper-catalyzed C–N bond coupling between 2-halobenzoates and *o*-phenylenediamines leading to a key intermediate that undergoes an intramolecular *N*-acylation to afford the corresponding dibenzodiazepinone structure in high yields (Scheme 1b) [14]. Another innovative strategy was reported by Laha et al., aiming to access dibenzodiazepinone structures via a double *N*-arylation of 2-aminobenzamides with 1,2-dihaloarenes using a palladium-based catalytic system [15].

Mechanistic investigations supported the fact that the regioselective *N*-arylation of the 2-aminobenzamide occurs first at the amide position. This approach enabled the successful synthesis of a broad spectrum of dibenzodiazepinone units in a one-pot fashion. The synthetic utility of Laha’s approach was highlighted by preparing the corresponding dibenzodiazepinone which was further reacted with *N*-methylpiperazine in the presence of TiCl_4_ to afford clozapine, an antipsychotic drug.

We now disclose a different, but facile approach to access several 10,11-dihydro-5*H*-dibenzo[*b*,*e*][1,4]diazepinones, using a sequential Chan–Lam and Buchwald–Hartwig intramolecular aminocarbonylation (Scheme 1d). This approach has not been reported previously (the methods a–c indicated in Scheme 1 have a different route, and none involve either a Chan–Lam or carbonylative cyclization). For the sake of health and safety, and given that our infrastructures did not permit the use of molecular CO, we felt more secure with a suitable surrogate. Not only that, the use of safer to use surrogates, is important for use in enabling technologies, like continuous flow and microwave-heated reactions. In fact, CO-free aminocarbonylation reactions are well known, and molybdenum hexacarbonyl (Mo(CO)_6_) is a very useful surrogate having been used in a multitude of reactions [16–17].

The present approach enables the formation of two C–N bonds along with a C–C bond and provides a good alternative to previously reported strategies, as it enables the formation of these structures in a multicomponent fashion in the presence of a CO surrogate through the in situ formation of an *o*-(2-bromophenyl)aminoaniline intermediate (Scheme 1d). It should be noted these target compounds have been of great interest to our group and in 2015 we reported a proposed novel methodology for the synthesis of dibenzodiazepines [18], however, upon later careful review of the product structure it was revealed that the purported dibenzodiazepine products were, in fact, diarylimines, which resulted from a nucleophilic addition of the aniline reagents to the aldimine substrates, followed by elimination of an tosylamine product. This was one of the principle driving forces for the development of the work discussed in this report.

## Results and Discussion

### Synthesis of *o*-(2-bromophenyl)aminoaniline via Buchwald–Hartwig C–N coupling

#### One-pot synthesis of dibenzodiazepinones

Our preliminary attempt to synthesize DBDAPs via B–H amination and carbonylation was carried out in the presence of *o*-phenylenediamine (**1a**) and 1,2-dibromobenzene (**2**) as model reactants using Pd(OAc)_2_ in combination with *t*-BuXPhos (2-di-*tert*-butylphosphino-2',4',6'-triisopropyl-1,1'-biphenyl) (5 mol %), and Et_3_N (2.5 equiv) as base in DMF. In this case, DMF served as the CO surrogate, as it was disclosed that DMF, the reaction solvent, could act as a potential carbon monoxide surrogate under certain circumstances, notably, in metal-catalyzed aminocarbonylation procedures [19–20]. Unfortunately, no DBDAP was obtained and we only observed the formation of intermediate **3a** in 25% yield (entry 1, Table 1). Next, the same procedure was carried out in the presence of molybdenum hexacarbonyl (Mo(CO)_6_, 2 equiv) as CO surrogate, under the previous conditions, but again we only observed the formation of intermediate **3a** in 21% yield (entry 2, Table 1). Changing the ligand to triphenylphosphine, did not provide any improvement of the reaction outcome as only traces of the intermediate **3a** were obtained (entry 3, Table 1). Switching to DBU as the base under these conditions, gave intermediate **3a** in 35% yield (entry 4, Table 1). In fact, DBU was previously shown by Wannberg and Larhed to be an effective ligand in a variety of highly efficient aminocarbonylation reactions with Mo(CO)_6_ due to its strong basicity and accelerated release of CO from this reagent [21]. The reaction was then screened using two different bidentate ligands, XPhos and XantPhos, and using the previous reaction conditions. However, we only obtained traces of intermediate **3a** (entries 5 and 6, Table 1). A slight improvement of the yield of the intermediate **3** was obtained when using DBU in dioxane, which gave **3a** in 42%, but only traces of the target DBDAP were observed (entry 7, Table 1). The difficulty encountered in the formation of DBDAP, prompted us to test alternative CO surrogates. When the reaction was performed using Co_2_(CO)_8_ (0.3 equiv) in the presence of DBU, the intermediate **3a** was isolated in 35% yield, but again no DBDAP **4** was obtained (entry 8, Table 1). Formic acid, an effective CO surrogate [20,22], was also screened. The reaction was carried out in the presence of acetic anhydride (Ac_2_O) as an activator, but unfortunately, no DBDAP was obtained (entry 9, Table 1). The use of triphosgene as the CO transfer agent failed to give any product.

**Table 1:** Explorative study of the sequential Buchwald–Hartwig amination/Pd-catalyzed carbonylative cyclization leading to DBDAPs.^a^

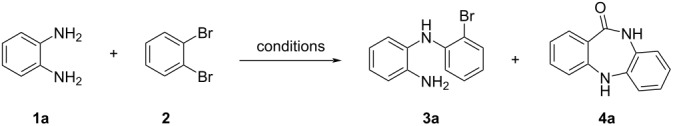							

Entry	Cat (mol %)	Ligand (5 mol %)	CO	Base	Solvent	**3a** ^b^	**4a** ^b^

1	Pd(OAc)_2_ (5)	*t-*BuXPhos	–	Et_3_N	DMF	25%	0
2	Pd(OAc)_2_ (5)	*t-*BuXPhos	Mo(CO)_6_	Et_3_N	DMF	21%	0
3	Pd(OAc)_2_ (5)	PPh_3_	Mo(CO)_6_	Et_3_N	DMF	traces	0
4	Pd(OAc)_2_ (5)	*t-*BuXPhos	Mo(CO)_6_	DBU	DMF	35%	0
5	Pd(OAc)_2_ (5)	XPhos	Mo(CO)_6_	Et_3_N	DMF	traces	0
6	Pd(OAc)_2_ (5)	XantPhos	Mo(CO)_6_	Et_3_N	DMF	traces	0
7^c^	Pd(OAc)_2_ (5)	*t-*BuXPhos	Mo(CO)_6_	DBU	dioxane	42%	traces
8^c^	Pd(OAc)_2_ (5)	*t-*BuXPhos	Co_2_(CO)_8_	DBU	DMF	35%	–
9^c^	Pd(OAc)_2_ (5)	*t-*BuXPhos	HCOOH/Ac_2_O	Et_3_N	DMF	–	–

^a^Reaction conditions: *o*-phenylenediamine (**1a**, 0.46 mmol), dibromobenzene (**2**, 0.46 mmol), Pd(OAc)_2_, base (2.5 equiv), CO surrogate (Mo(CO)_6_ and other CO surrogate (2 equiv) or Co_2_(CO)_8_ (0.3 equiv)), solvent (5 mL), 130 °C, 20 h. ^b^Isolated yields. ^c^The reaction was carried out during 24 h.

**Scheme 2:**
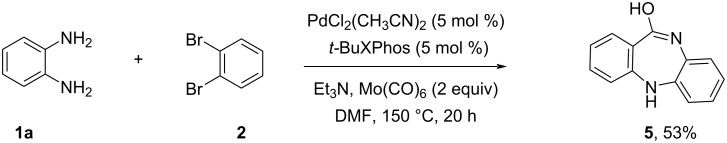
One-pot synthesis of 5*H*-dibenzo[*b*,*e*][1,4]diazepin-11-ol (**5**).

When we undertook the B–H amination/carbonylative cyclization of *o*-phenylenediamine (**1a**) with 1,2-dibromobenzene (**2**) in the presence of 5 mol % of PdCl_2_(CH_3_CN)_2_ and 5 mol % of *t-*BuXPhos, with Et_3_N (2.5 equiv) and Mo(CO)_6_ in DMF at 150 °C, surprisingly this afforded the 5*H*-dibenzo[*b*,*e*][1,4]diazepin-11-ol (**5**), the tautomer of DBDAP (**4a**) in 53% (Scheme 2). We attempted to convert compound **5** into the keto form **4a** by using TFA to shift the equilibrium towards the desired product, but this proved to be futile under these conditions, as only the iminol **5** structure was observed.

#### Attempt at accessing dibenzodiazepinone via step-wise synthesis

Due to the difficulty encountered in the one-step synthesis of DBDAPs, we embarked on an in-depth study of the B–H coupling/carbonylative cyclization in a step-wise fashion. Our first attempt was conducted using the previous conditions, which led to the desired compound **3a** in 15% yield (entry 1, Table 2). Changing the ligand to PPh_3_ under the same conditions (entry 2, Table 2) resulted in poorer results, as only traces of the desired compound **3a** were observed. Then, we considered XPhos (entry 3, Table 2), and the bidendate ligands XantPhos and DPEPhos (entries 4 and 5, Table 2), but no improvements were observed. Then, we tested an alternative palladium source, namely Pd_2_dba_3_, but again only traces of the compound **3a** were observed (entry 6, Table 2). Next, we increased the Pd(OAc)_2_ catalyst loading to 10 mol % and the amount of the *t-*BuXphos ligand to 15 mol % in the presence of DBU and DMF. Under these conditions, the intermediate **3a** was obtained in 15% yield along with the undesired dihydrophenazine **6** side product in 5% yield, produced by a further C–N bond coupling (entry 7, Table 2). We decided to decrease the amount of base and time, but little improvement was observed (entry 8, Table 2). Given the well-established role of the base on the B–H coupling, we considered exploring alternative bases. We conducted the reaction in the presence of the previously disclosed catalytic system but using *t*-BuOK as the base and obtained compound **3a** in 22% yield (entry 9, Table 2). Conducting the reaction in the presence of Cs_2_CO_3_ in DMF, failed to provide any improvement (entry 10, Table 2). However, replacing DMF by dioxane as the solvent in the presence of DBU led to a significant improvement in the yield of the reaction, as the intermediate **3a** could be obtained in 40% yield, along with the phenazine **6** in 48% (entry 11, Table 2). Next, we screened other ligands such as XPhos and DPEPhos in the presence of Cs_2_CO_3_ as base in dioxane, however, the undesired phenazine product **6** was still obtained in moderate yield under these conditions (entries 12 and 13, Table 2). In the presence of SPhos as ligand, Cs_2_CO_3_, and toluene as solvent, the desired intermediate **3a** was obtained in 20% yield along with 55% of the phenazine **6** (entry 14, Table 2). Although toluene was shown to be a good solvent for this B–H coupling reaction, we were unable to prevent the double B–H reaction from occurring leading to the phenazine **6**, even when shortening the reaction time to 1 h.

**Table 2:** Influence of the catalytic system, base, and solvent combination on the outcome of the Buchwald–Hartwig reaction.^a^

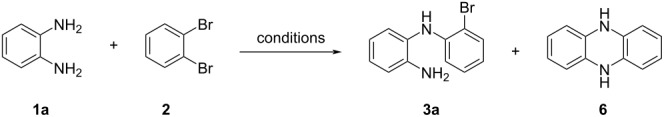							

Entry	Cat (mol %)	Ligand	Base	Solvent	Time	**3a** ^b^	**6** ^b^

1	Pd(OAc)_2_ (5)	*t-*BuXPhos	Et_3_N	DMF	10 h	15	–
2	Pd(OAc)_2_ (5)	PPh_3_	Et_3_N	DMF	10 h	–	–
3	Pd(OAc)_2_ (5)	XPhos	Et_3_N	DMF	10 h	–	–
4	Pd(OAc)_2_ (5)	XantPhos	Et_3_N	DMF	10 h	–	–
5	Pd(OAc)_2_ (5)	DPEPhos	Et_3_N	DMF	10 h	–	–
6	Pd_2_dba_3_ (5)	*t-*BuXPhos	DBU	DMF	24 h	traces	–
7	Pd(OAc)_2_ (10)	*t-*BuXPhos	DBU	DMF	24 h	15	5
8^c^	Pd(OAc)_2_ (10)	*t-*BuXPhos	DBU	DMF	10 h	10	–
9^c^	Pd(OAc)_2_ (10)	*t-*BuXPhos	*t*-BuOK	DMF	24 h	22	–
10^c^	Pd(OAc)_2_ (10)	*t-*BuXPhos	Cs_2_CO_3_	DMF	24 h	traces	–
11^c^	Pd(OAc)_2_ (10)	*t-*BuXPhos	DBU	dioxane	24 h	40	48
12^c^	Pd(OAc)_2_ (10)	XPhos	Cs_2_CO_3_	dioxane	10 h	45	39
13^d^	Pd(OAc)_2_ (10)	DPEPhos	Cs_2_CO_3_	dioxane	10 h	35	30
14^d^	Pd(OAc)_2_ (10)	SPhos	Cs_2_CO_3_	toluene	10 h	20	55

^a^Reaction conditions: *o*-phenylenediamine (**1a**, 0.46 mmol), dibromobenzene (**2**, 0.46 mmol), Pd catalytic system, base (2.5 equiv), DMF (5 mL), 110 °C, 24 h; *o*-(2-bromophenyl)aminoaniline (**3a**) and 5,10-dihydrophenazine (**6**) products were detected by TLC and ^1^H NMR, and yields determined after product isolation. ^b^Isolated yields. ^c^1.5 Equivalents of base were used. ^d^1.2 Equivalents of base were used.

Interestingly, we noticed that in the initial one-pot reactions indicated in Table 1, the yields of the diarylamine **3a** were better when Mo(CO)_6_ was present (compare entry 4, Table 1 to entry 7, Table 2). This might be due to (a) the fact that the temperature used for the reactions described in Table 1, involving Mo(CO)_6_ were 20 °C higher than the reactions described in Table 2 and/or (b) Mo(CO)_6_ acts as a co-catalyst. An investigative study was thus undertaken to elucidate the effect of the molybdenum reagent on the reaction using a simple model system consisting of aniline and bromobenzene (see Figure S1 and Table S1 in Supporting Information File 1). The experimental design was limited, in that we only monitored the reaction over a 90 min period. Contrary to what was originally believed the reaction without the Mo reagent gave better results during the first 90 min, but without additional data it is impossible to draw firm conclusions about this reaction, and thus further studies will be carried out in the near future.

After uncovering the optimum conditions to access *o*-(2-bromophenyl)aminoaniline via the B–H coupling reaction, it was decided to explore the carbonylative intramolecular cyclization of the intermediate **3a** using different catalytic systems. To elucidate the role of the palladium catalyst in this process, we carried out the initial attempt under metal free-conditions using molybdenum hexacarbonyl (Mo(CO)_6_) as CO surrogate, in the presence of Et_3_N in DMF. The reaction was performed at 130 °C, as we believed that high temperature will promote the cyclization of the sterically hindered intermediate **3a**, but no DBDAP was achieved under these conditions (entry 1, Table 3). Next, Pd(OAc)_2_ was employed under ligand-free conditions, but again the desired DBDAP product **4a** could not be attained (entry 2, Table 3). Then, we performed the reaction in the presence of *t-*BuXPhos as ligand and in this case, only traces of the DBDAP **4a** were obtained (entry 3, Table 3). When the reaction was carried out in the presence of DPEPhos (entry 4, Table 3), we were delighted to obtain the final dibenzodiazepine in 80% yield. Next, we tested another bidentate ligand, XantPhos, which led to the obtention of the desired product **4a** in an excellent yield of 90% (entry 5, Table 3). This result implied that diphosphine ligands were essential for the success of this reaction. The reactivity of Co_2_CO_8_ as CO surrogate was also explored, in this case the reaction afforded the DBDAP product **4a** in 55% yield (entry 6, Table 3). Molybdenum hexacarbonyl (Mo(CO)_6_), was shown to be a powerful CO surrogate in this carbonylative intramolecular cyclization. The efficacy of Mo(CO)_6_ is due to the energetically favorable dissociation of Mo(CO)*_n_* into Mo(CO)*_n_*_−1_ which was proven to be a highly exothermic reaction in the presence of metal catalysts especially after the dissociation of the first CO group [23].

**Table 3:** The intramolecular catalytic carbonylative cyclization conditions for *o*-(2-bromophenyl)aminoaniline.^a^

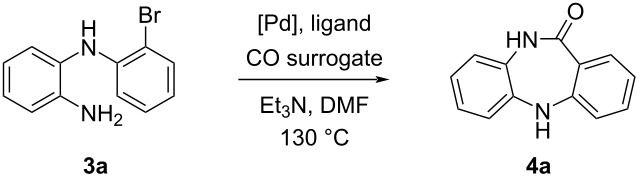			

Entry	Catalytic system	CO surrogate	Yield **4a**^b^ (over 2 steps)

1	None	Mo(CO)_6_	–
2	Pd(OAc)_2_	Mo(CO)_6_	–
3	Pd(OAc)_2_/*t-*BuXPhos	Mo(CO)_6_	traces
4	Pd(OAc)_2_/DPEPhos	Mo(CO)_6_	80% (44%)
5	Pd(OAc)_2_/XantPhos	Mo(CO)_6_	90% (50%)
6	Pd(OAc)_2_/XantPhos	Co_2_(CO)_8_	55% (30%)

^a^Reaction conditions: *o*-(2-bromophenyl)aminoaniline (**3a**, 0.46 mmol), Pd(OAc)_2_ (10 mol %), ligand (30 mol %, Mo(CO)_6_ (1 equiv), Et_3_N (1 equiv) DMF (5 mL), 130 °C, 20 h. The reaction was monitored by TLC. ^b^Isolated yields.

It should be noted that the best overall yield for the synthesis of **4a** using the step-wise approach was 50% (Table 3).

### Synthesis of dibenzodiazepinones via Chan–Lam amination/carbonylative coupling

#### Synthesis of *o*-(2-bromophenyl)aminoaniline via Chan–Lam C–N coupling

Inspired by the previously independently reported work by Chan [24] and Lam [25] and co-workers, we considered performing the reaction of *o*-phenylenediamine (**1a**) with 2-bromophenylboronic acid (**7**) in the presence of Cu(OAc)_2_, Et_3_N as base in DCM at 50 °C (entry 1, Table 4), and gratifingly under these conditions, the reaction afforded compound **3a** in 48% yield. The Chan–Lam couplings were undertaken under an aerobic atmosphere which is an environmentally benign oxidant. Further screening using dioxane as solvent resulted in an increase in the yield to 55%, whilst DMF gave access to **3a** in a lower yield (30%) (entries 2 and 3, Table 4). Next, we considered testing the performance of copper iodide (CuI, 20 mol %) as catalyst, in the presence of Et_3_N both in dioxane and DMF. These conditions resulted in the obtainment of the desired compound **3a** in 59% and 35% yield, respectively (entries 4 and 5, Table 4). Increasing the reaction temperature to 100 °C resulted in a reduction of the reaction yield due to purported catalyst degradation (entry 6, Table 4). Then, we considered decreasing the amount of CuI to 10 mol % which led to a decrease of the reaction yield to 31% (entry 7, Table 4). Other bases such as dimethylaminopyridine (DMAP) and diisopropylethylamine (DIPEA) were tested under the previous reaction conditions but failed to improve the yield. In the presence of DMAP, no sign of the target compound was detected, while with DIPEA, **3a** was obtained in 19% yield (entries 8 and 9, Table 4). Then, we tested CuSO_4_·5H_2_O in the presence of Et_3_N as the base in two different solvents DCM and dioxane, and both gave the desired compound in good yields of 60% and 50%, respectively (entries 10 and 11, Table 4).

**Table 4:** Optimization of the Chan–Lam coupling conditions between *o*-phenylenediamine (**1a**) and 2-bromophenylboronic acid (**7**).^a^

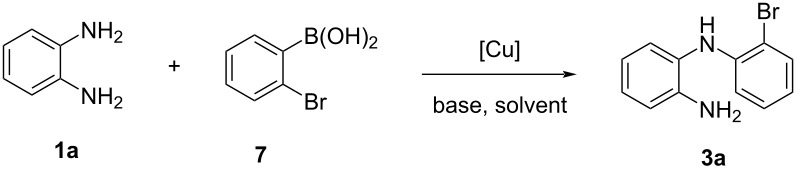				

Entry	Copper source	Base	Solvent	Yield **3a**^b^

1	Cu(OAc)_2_	Et_3_N	DCM	48%
2	Cu(OAc)_2_	Et_3_N	dioxane	55%
3	Cu(OAc)_2_	Et_3_N	DMF	30%
4	CuI	Et_3_N	dioxane	59%
5	CuI	Et_3_N	DMF	35%
6^c^	CuI	Et_3_N	DMF	23%
7^d^	CuI	Et_3_N	DMF	31%
8	CuI	DMAP	DMF	ND
9	CuI	DIPEA	DMF	19%
10	CuSO_4_·5H_2_O	Et_3_N	dioxane	60%
11	CuSO_4_·5H_2_O	Et_3_N	DCM	50%

^a^Reaction conditions: *o*-phenylenediamine (**1a**, 0.46 mmol), 2-bromophenylboronic acid (**7**, 0.46 mmol, 1 equiv), copper catalyst (20 mol %), base (1.5 equiv), solvent (5 mL), 50 °C, 1–2 h. The reaction was monitored by TLC. ^b^Isolated yield; ND: not detected ^c^Reaction performed at 100 °C. ^d^10 mol % CuI were used.

#### Accessing the scope of the one-pot Chan–Lam/Pd-catalyzed carbonylative cyclization

Once the abovementioned conditions were obtained, we undertook a screening of the Chan–Lam/carbonylative synthesis of the DBDA in a one-pot manner. In the first reaction, *o*-phenylenediamine (**1a**) and 2-bromophenylboronic acid (**7**) were reacted in a pressure flask under an inert atmosphere using copper iodide (CuI) as the copper catalyst, Et_3_N, and Mo(CO)_6_ as CO surrogate in the presence of Pd(OAc)_2_/XantPhos as catalytic system for the carbonylative intramolecular cyclization. These conditions led to the obtainment of the compound **4a** in 45% yield (entry 1, Table 5). Using dioxane as solvent, in the presence of the same catalytic system, afforded the desired structure **4a** in moderate yield (37%, entry 2, Table 5). Changing the ligand to DPEPhos resulted in a slight increase of the yield of **4a**, which was obtained in 40% (entry 3, Table 5). When the catalytic system PdCl_2_(NCCH_3_)/DPEPhos was employed, the product **4a** was accessed in a lower yield of 18% (entry 4, Table 5). Under these reaction conditions, copper bromide was shown to be a good catalyst for this transformation, as it allowed the production of **4a** in 40% yield (entry 5, Table 5). Then, we considered testing the performance of a different copper source Cu(OAc)_2_ under these reaction conditions using DMF and dioxane as solvents. In these cases, the final compound **4a** was obtained in 38% and 41% yield, respectively (entries 6 and 7, Table 5). Next, we evaluated the performance of CuSO_4_·5H_2_O in dioxane and DMF, but lower yields were obtained (entries 8 and 9, Table 5). With the optimized conditions in hand, we tested the one pot Chan–Lam intramolecular cyclization with several other *o*-phenyldiamine derivatives, however, several impurities were obtained. In the hope of obtaining better yields (best obtained with the one pot method = 41%) we looked at the step-wise synthesis.

**Table 5:** Substrate scope of the one-pot synthesis of dibenzodiazepine using via Chan–Lam coupling/carbonylative intramolecular cyclization.^a^

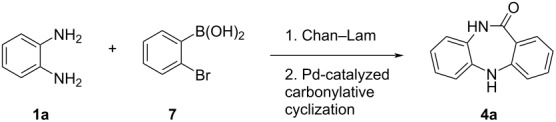					

Entry	Copper source	Base	Solvent	Pd/ligand	Yield **4a**^b^

1	CuI	Et_3_N	DMF	Pd(OAc)_2_/XantPhos	45%
2	CuI	Et_3_N	dioxane	Pd(OAc)_2_/XantPhos	37%
3	CuI	Et_3_N	DMF	Pd(OAc)_2_/DPEPhos	40%
4	CuI	Et_3_N	DMF	PdCl_2_(NCCH_3_)_2_/XantPhos	18%
5	CuBr	Et_3_N	DMF	Pd(OAc)_2_/Xantphos	40%
6	Cu(OAc)_2_	Et_3_N	DMF	Pd(OAc)_2_/DPEPhos	38%
7	Cu(OAc)_2_	Et_3_N	dioxane	Pd(OAc)_2_/DPEPhos	41%
8	CuSO_4_.5H_2_O	Et_3_N	dioxane	Pd(OAc)_2_/DPEPhos	18%
9	CuSO_4_.5H_2_O	Et_3_N	DMF	Pd(OAc)_2_/DPEPhos	traces

^a^Reaction conditions: *o*-phenylenediamine (**1a**, 0.5 mmol), **7** (0.5 mmol), Cu catalyst (20 mol %), base (0.6 mmol), solvent (5 mL), 50 °C, 1 h; Pd catalytic system (5 mol %), Mo(CO)_6_ (0.5 mmol) and base (0.5 mmol), 130 °C. ^b^Isolated yields.

#### Synthesis of 5,10-dihydro-11*H*-dibenzo[*b*,*e*][1,4]diazepin-11-ones via a stepwise Chan–Lam/carbonylative cyclization

**Scheme 3:**
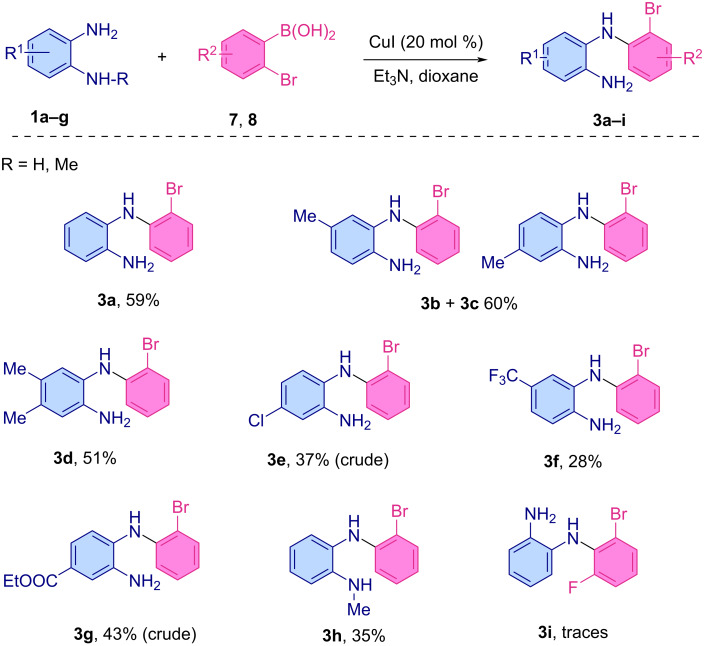
Scope of the Chan–Lam coupling between *o*-phenylenediamines and 2-bromophenylboronic acids (please note products **3e** and **3g** contained some unidentified impurities that were impossible to remove via chromatography).

After disclosing the optimal conditions for the Chan–Lam coupling, we screened different varieties of *o*-phenylenediamine derivatives. Overall, the *o*-phenylenene substrates bearing electron-donating substituents on the benzene ring proceeded smoothly under these conditions and led to the desired structures in moderate to good yields (Scheme 3). The reactions were generally regioselective, except in the case of 4-methyl-*o*-phenylenediamine (**1b**) and *o*-bromophenylboronic acid (**7**) which gave a mixture of products **3b** and **3c** in 60% yield (the ratio could not be determined). These were eventually separated and used in the cyclization step discussed below. The dimethylated *o*-phenylenediamine **1d**, gave the desired compound **3d** in 51% yield. A slight decrease in yield was observed in the presence of an ester (COOEt) substituent, which furnished compound **3g** in 43% yield. Lower yields were observed in the case of electron-withdrawing substituents such as Cl and CF_3_ groups, which afforded the compounds **3e** and **3f** in 37% and 28% yields, respectively. The N-methylated precursor **1h** was also tolerated by this system and afforded the desired compound **3h** in 35% yield. Unfortunately, the reaction with 2-bromo-6-fluorophenylboronic acid (**8**) afforded the corresponding product **3i** only in trace amounts.

**Scheme 4:**
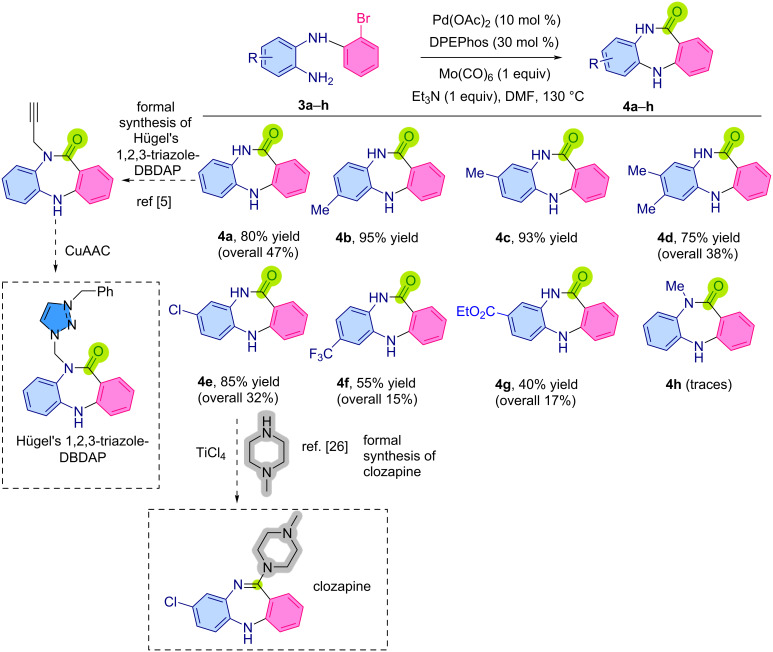
Scope of the synthesis of DBDAPs. Please note that product **4g** contained some unidentified impurities that were impossible to remove via chromatography.

With the *o*-(2-bromophenyl)aminoaniline derivatives in hand, we conducted the carbonylative intramolecular cyclization according to the previously disclosed conditions, in order to access the desired DBDAP structures. The unsubstituted DBDAP structure **4a** was obtained in 80% under these conditions (Scheme 4). The methyl-substituted *o*-(2-bromophenyl)aminoanilines **3** obtained via Chan–Lam coupling could be efficiently separated and were subjected to the intramolecular carbonylative cyclization to yield the DBDAPs **4b** and **4c** in excellent yields of 95% and 93%, respectively. The dimethyl-DBDAP **4d** could also be efficiently obtained under these conditions in 75% yield. The chloro-substituted DBDAP **4e**, which is the intermediate to the antipsychotic drug clozapine, could also be obtained in good yield (this represented a formal synthesis to clozapine [26], if the procedure of Rao [27] is used, which entails heating **4e** with 1-methylpiperidine and Ti(IV)Cl_4_, Scheme 4). Also compound **4a** can be transformed to Hügel's 1,2,3-triazole-DBDAP using the methodology described in their report (Scheme 4) [5]. The CF_3_- and COOEt-substituted DBDAs **4f** and **4g** were obtained with a slightly decreased yield of 55% and 40%, respectively. The *N*-methyldibenzodiazepine **4h** could be accessed, but only in trace quantity. It should be noted that the stepwise approach was slightly better than the one-pot approach in the case of the synthesis **4a** (47% vs 41%).

**Scheme 5:**
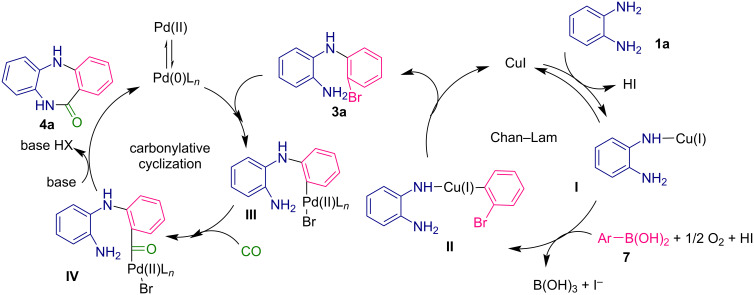
Proposed mechanism.

Our mechanistic proposal is based on the information in previous reports by the groups of Bose [28], Watson [29], and Stahl [30]. Mechanistically, under basic conditions, the reaction is triggered by copper-catalyzed activation of *o*-phenylenediamine (**1a**), followed by the oxygen-promoted insertion of the phenylboronic acid coupling partner **7** to deliver intermediate **II** that undergoes reductive elimination to give diarylamine **3a** along with regeneration of the copper catalyst (Scheme 5). Then, a palladium-promoted oxidative addition of the C–Br bond takes places to deliver palladium species **III**. Then the insertion of CO that is released by Mo(CO)_6_, should afford intermediate **IV** that undergoes a base-promoted intramolecular cyclization via nucleophilic attack of the amine [31]. Finally, the dibenzodiazepinone **4a** would be obtained through reductive elimination of the palladium catalyst.

## Conclusion

In summary, we have reported two one-pot pathways and two step-wise pathways to access dibenzodiazepinone (DBDAP) derivatives via copper-catalyzed Chan–Lam amination/carbonylative cyclization and Buchwald–Hartwig amination/carbonylative cyclization and their step-wise counterparts. Although the one-pot method worked for one example in both cases (but in one case it gave the DBDAP enol form), it failed to work for other substrates, and for that reason we had to rely on the step-wise approach. The more efficient method to access the diamine intermediates **3** was via the Chan–Lam amination (milder conditions, cheaper, earth-abundant catalyst, no expensive ligand requirement) as the Buchwald–Hartwig amination required harsher conditions and an expensive metal catalyst, and also gave an unwanted phenazine side product. The sequential stepwise Chan–Lam amination/carbonylative cyclization afforded a number of DBDAP products, showing good functional group tolerance and giving the final products in good yields. In terms of the overall best efficiency, it also would appear that the step-wise Chan–Lam/Pd-catalyzed carbonylative cyclization was slightly better than the one-pot method. The most important of which was the chloro-containing DBDAP **4e** that can be used to synthesize the antipsychotic drug clozapine (see above), a triazole-hybrid with anticancer properties, and can easily be used as the key part in the synthesis of other drugs like dibenzepine and biologically active natural products such as BU-4664L. We are currently looking at this methodology to access some of these targets, including the agrochemical boscalid.

## Experimental

### Synthesis of *o*-(2-bromophenyl)aminoaniline (**3a**)

**Via Buchwald–Hartwig coupling:***o*-Phenylenediamine (**1a**, 0.05g, 1 equiv, 0.46 mmol) was added to a Radleys reaction tube (a Radleys^®^ 12 position carousel reactor station was used) under N_2_ and dissolved in dry dioxane (5 mL). Next, (0.055 mL, 0.46 mmol) of 1,2-dibromobenzene (**2**) was added to the reaction mixture, followed by the addition of Pd(OAc)_2_ (0.01 g, 0.046 mmol), XPhos (0.032 g, 0.069 mmol), and Cs_2_CO_3_ (0.18 g, 0. 56 mmol, 1,2 equiv). The resulting reaction mixture was allowed to stir at 100 °C. The reaction was left stirring for several hours, followed by TLC. After consumption of the starting material (verified through TLC). The reaction was allowed to cool and filtered through a celite pad to remove the residual catalyst and base. The solvent was then evaportated under reduced pressure and the crude was purified by flash chromatography (hexane/AcOEt 9:1), to yield *o*-(2-bromophenyl)aminoaniline (**3**) compound as a purple oil (0.057 g, 47% yield).

**Via Chan–Lam coupling:***o*-Phenylenediamine (**1a**, 0.05 g, 1 equiv, 0.46 mmol) was added to a round-bottomed flask and dissolved in dry dioxane (5 mL). Next, 2-bromophenyl)boronic acid (**7**, 0.092 g, 1 equiv,0.46 mmol) was added, followed by the addition of Et_3_N (0.07 mL, 0.055 mmol), CuI (0.018 g, 0.092 mmol, 20 mol %), and molecular sieves 3 Å. The reaction was left stirring at room temperature for several hours, and monitored by TLC. After consumption of the starting material (verified through TLC), the reaction mixture was filtered through a celite pad to remove the residual catalyst and molecular sieves. The volatiles were then evaporated under reduced pressure and the crude was purified by flash chromatography (hexane/AcOEt 9:1), to yield *o*-(2-bromophenyl)aminoaniline (**3a**) as a purple oil (0.07 g, 59% yield).^1^H NMR (CDCl_3_, 400 MHz) δ 4.00 (s, NH_2_, 2H), 5.76 (s, NH, 1H), 6.59–6.61 (d, *J* = 8 Hz, Ar, 1H), 6.65–6.69 (t, *J* = 8 Hz, Ar, 1H), 6.79–6.83 (t, *J* = 8 Hz, Ar, 1H), 6.85–6.87 (d, *J* = 8 Hz, Ar, 1H), 7.09–7.13 (m, Ar, 3H), 7.49–7.51 (d, *J* = 8 Hz, Ar, 1H); ^13^C NMR (CDCl_3_, 100 MHz) δ 110.42, 114.41, 116.47, 119.48, 119.70, 127.00, 127.04, 128.39, 132.62, 142.45, 143.03; HRESIMS (*m*/*z*): [M + H^+^] calcd for C_12_H_11_BrN_2_, 263,0184; found, 263.0178.

### Synthesis of 5,10-dihydro-11*H*-dibenzo[*b*,*e*][1,4]diazepin-11-one (**4a**)

*o*-(2-Bromophenyl)aminoaniline (**3a**, 0.05 g, 0.19 mmol) was added to a Radley’s^®^ 12 position carousel reactor tube to which DMF, then Pd(OAc)_2_ (4.26 mg, 0.019 mmol), DPEPhos (30 mg, 0.057 mmol), Mo(CO)_6_ (50 mg, 1 equiv, 0.19 mmol), and Et_3_N (0.026 mL, 0.19 mmol) were added. The reaction mixture was then stirred at 130 °C under a nitrogen atmosphere. After completion of the reaction, as determined by TLC, the reaction mixture was allowed to cool to room temperature. The mixture was filtered through a pad of celite and washed with DCM, then, the solvent was evaporated under reduced pressure to give a crude mixture. Further purification by flash chromatography (hexane/AcOEt 1:1), gave the desired compound 5,10-dihydro-11*H*-dibenzo[*b*,*e*][1,4]diazepin-11-one (**4a**) as a yellow solid yield (0.032 g, 80%). Mp 249–251 °C; ^1^H NMR (DMSO-*d*_6_, 400 MHz) δ 6.87–7.00 (m, Ar, 6H), 7.31–7.35 (t, *J* = 8 Hz, Ar, 1H), 7.66–7.68 (d, *J* = 8 Hz, Ar,1H), 7.84 (s, Ar, 1H), 9.85 (s, Ar, 1H); ^13^C NMR (CDCl_3_, 100 MHz) δ 119.52, 120.23, 121.17, 121.73, 123.24, 123.40, 124.95, 130.29, 132.56, 133.67, 140.43, 150.92, 168.40; ESIMS (*m*/*z*): 221.12 [M + H^+^].

## Supporting Information

File 1 Experimental procedures and spectral data (NMR, mass spectra) and key kinetic studies.

## References

[1] Khokhar J Y, Henricks A M, Sullivan E D K, Green A I (2018). Adv Pharmacol (San Diego, CA, U S).

[2] Jafari S, Fernandez‐Enright F, Huang X-F (2012). J Neurochem.

[3] Cao K, Yan J, Yan F, Yin T (2021). Mol Diversity.

[4] Miyanaga S, Sakurai H, Saiki I, Onaka H, Igarashi Y (2010). Bioorg Med Chem Lett.

[5] Praveen Kumar C, Reddy T S, Mainkar P S, Bansal V, Shukla R, Chandrasekhar S, Hügel H M (2016). Eur J Med Chem.

[6] Minucci S, Pelicci P G (2006). Nat Rev Cancer.

[7] De Clercq D J H, Heppner D E, To C, Jang J, Park E, Yun C-H, Mushajiang M, Shin B H, Gero T W, Scott D A (2019). ACS Med Chem Lett.

[8] Aniban X, Mamidala S, Burke A J (2018). Eur J Org Chem.

[9] Guo W, Zhao M, Tan W, Zheng L, Tao K, Fan X (2019). Org Chem Front.

[10] Chen J-Q, Li J-H, Dong Z-B (2020). Adv Synth Catal.

[11] Schoenberg A, Heck R F (1974). J Org Chem.

[12] Brennführer A, Neumann H, Beller M (2009). Angew Chem, Int Ed.

[13] Tsvelikhovsky D, Buchwald S L (2011). J Am Chem Soc.

[14] Zhang Q-Y, Wang X-J, Tian Y-L, Qi J-G, Li C, Yin D-L (2013). Chin Chem Lett.

[15] Laha J K, Manral N, Hunjan M K (2019). New J Chem.

[16] Åkerbladh L, Odell L R, Larhed M (2019). Synlett.

[17] Odell L R, Russo F, Larhed M (2012). Synlett.

[18] Peixoto D, Locati A, Marques C S, Goth A, Ramalho J P P, Burke A J (2015). RSC Adv.

[19] Wan Y, Alterman M, Larhed M, Hallberg A (2002). J Org Chem.

[20] Oseghale C O, Onisuru O R, Fapojuwo D P, Mogudi B M, Molokoane P P, Maqunga N P, Meijboom R (2021). RSC Adv.

[21] Wannberg J, Larhed M (2003). J Org Chem.

[22] Hussain N, Chhalodia A K, Ahmed A, Mukherjee D (2020). ChemistrySelect.

[23] Dupont C, Wan X, Petukhov M, Krüger P (2014). Int J Quantum Chem.

[24] Chan D M T (1996). Tetrahedron Lett.

[25] Lam P Y S, Clark C G, Saubern S, Adams J, Winters M P, Chan D M T, Combs A (1998). Tetrahedron Lett.

[26] Nucifora F C, Mihaljevic M, Lee B J, Sawa A (2017). Neurotherapeutics.

[27] Venkat Rao S (2020). Arabian J Chem.

[28] Bose S, Dutta S, Koley D (2022). ACS Catal.

[29] Vantourout J C, Miras H N, Isidro-Llobet A, Sproules S, Watson A J B (2017). J Am Chem Soc.

[30] King A E, Ryland B L, Brunold T C, Stahl S S (2012). Organometallics.

[31] Shen C, Neumann H, Wu X-F (2015). Green Chem.

