# Supplementary material for: Metal-catalyzed coupling/carbonylative cyclizations for accessing dibenzodiazepinones: an expedient route to clozapine and other drugs
Source: Beilstein J Org Chem. 2024 Jan 31;20:193–204. doi: 10.3762/bjoc.20.19 (PMC10840530; doi:10.3762/bjoc.20.19)
Supplement: File 1 — Experimental procedures and spectral data (NMR, mass spectra) and key kinetic studies. [file Beilstein_J_Org_Chem-20-193-s001.pdf]

BEILSTEIN JOURNAL OF ORGANIC CHEMISTRY

Supporting Information

for

Metal-catalyzed coupling/carbonylative cyclizations for accessing dibenzodiazepinones: an expedient route to clozapine and other drugs

Amina Moutayakine and Anthony J. Burke

Beilstein J. Org. Chem. **2024,** *20,* 193–204. doi:10.3762/bjoc.20.19

Experimental procedures and spectral data (NMR, mass spectra) and key kinetic studies

License and Terms: This is a supporting information file under the terms of the Creative Commons Attribution License (https://creativecommons.org/licenses/by/4.0). Please note that the reuse, redistribution and reproduction in particular requires that the author(s) and source are credited and that individual graphics may be subject to special legal provisions.
The license is subject to the *Beilstein Journal of Organic Chemistry* terms and conditions: (https://www.beilstein-journals.org/bjoc/terms)

Table of contents

I) General experimental S1

II) Synthesis of *o*-(2-bromophenyl)aminoanilines S2

III) Synthesis of dibenzodiazepinones (DBDAs) S7

IV) Kinetic study of the molybdenum/palladium Buchwald-Hartwig coupling reaction S11

V) NMR spectra for *o*-(2-bromophenyl)aminoanilines........ S14

VI) Mass spectrum of *o*-(2-bromophenylamino)-*N*-methylaniline (**3h**) S21

VII) NMR spectra for 5,10-dihydro-11*H*-dibenzo[*b*,*e*][1,4]diazepin-11-ones S23

VIII) Mass spectrum of 10-methyl-5,10-dihydro-11*H*-dibenzo[*b*,*e*][1,4]diazepin-11-one (**4h**) ... S29

IX) References S29

General experimental

General considerations*:* Reagents were obtained from Sigma Aldrich, Acros, Strem and Alfa Aesar and were used as received. The solvents used were dried using standard laboratory techniques. The catalytic reactions were conducted in a Radley's 12-position carousel reactor under a nitrogen atmosphere or in round-bottomed flasks. Column chromatography was carried out on silica gel (Carlo Erba, 40–63 µm (flash) and 60–200 µm, 60A). Thin-layer chromatography (TLC) was carried out on aluminum-backed Kieselgel 60 F254 plates (Merck and Machery Nagel).

Plates were visualized either by UV light or with phosphomolybdic acid in ethanol. Melting points (mp) were determined with a Barnstead Electrothermal 9100 apparatus and are uncorrected. NMR spectra were recorded with a Bruker Avance III instrument (400 MHz) with a broad band probe. Chemical shifts are quoted in parts per million (ppm) relative to $\delta = 0.0$ ppm and were referenced to the appropriate non-deuterated solvent peak. Coupling constants (J) are reported in Hz and refer to apparent peak multiplicities. Splitting patterns are reported as s, singlet; d, doublet; t, triplet; m, multiplet; br, broad.

Low-resolution mass spectra (LRMS) were recorded with a quadrupole mass spectrometer Waters ZQ4000 and high-resolution mass spectra (HRMS) on a Thermo Orbitrap Q-exactive focus at a resolution of 70000 at the Chemistry Department, University of Salamanca (by Dr. César Raposo). ESI was used as ionization method, and the samples were dissolved in

methanol. In the case of the HRMS, an alternating method between positive and negative modes was applied and the mode with the best signal was used for the determination of the exact mass.

I. Synthesis of *o*-(2-bromophenyl)aminoanilines (3)

I.1 – Synthesis of *o*-(2-bromophenyl)aminoaniline (3a)

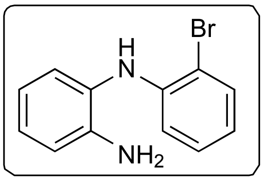

Via Buchwald–Hartwig coupling: *o*-phenylenediamine (**1a**, 0.05g, 1 equiv, 0.46 mmol) was added to a Radleys reaction tube (a Radleys® 12 position carousel reactor station was used) under N_2 and dissolved in dry dioxane (5 mL). Next, 1,2- dibromobenzene (**2**, 0.055 mL, 0.46 mmol) was added to the reaction mixture, followed by the addition of $Pd(OAc)_2$ (0.01 g, 0.046 mmol), XPhos (0.032 g, 0.069 mmol), and Cs_2CO_3 (0.18 g, 0.05 mmol). The resulting reaction mixture was allowed to stir at 100 ºC. The reaction was left stirring for several hours, followed by TLC. After consumption of the starting material (verified through TLC). The reaction was allowed to cool down, and was filtered through a celite pad to remove the residual catalyst and base. The solvent was then evaportated under reduced pressure and the crude was purified by flash chromatography (hexane/AcOEt 9:1, to yield the *o*-(2-bromophenyl)aminoaniline (**3**) compound as a purple oil (0.057 g, 47% yield).

Via Chan–Lam coupling: *o*-phenylenediamine (**1a**, 0.05 g, 1 equiv, 0.46 mmol) was added to a round-bottomed flask and dissolved in dry dioxane (5 mL). Next, 2-bromophenyl)boronic acid (**7**, 0.092 g, 1 equiv, 0.46 mmol) was added, followed by the addition of Et_3N (0.07 mL, 0.055 mmol), CuI (0.018 g, 0,092 mmol, 20 mol %), and molecular sieves 3 Å. The reaction mixture was left stirring at room temperature for several hours, and monitored by TLC. After consumption of the starting material (verified through TLC), the reaction mixture was filtered through a celite pad to remove the residual catalyst and molecular sieves. The solvent was then evaporated under reduced pressure and the crude was purified by flash chromatography (hexane/AcOEt 9:1), to yield the *o*-(2-bromophenyl)aminoaniline (**3a**) as a purple oil (0.07g, 59% yield).

^{1}H NMR ($CDCl_3$, 400 MHz) δ: 4.00 (s, NH_2, 2H), 5.76 (s, NH, 1H), 6.59-6.61 (d, *J*= 8Hz, Ar, 1H), 6.65-6.69 (t, *J*= 8Hz, Ar, 1H), 6.79-6-83 (t, *J*= 8Hz, Ar, 1H), 6.85-6.87 (d, *J*= 8Hz, Ar, 1H), 7.09-7.13 (m, Ar, 3H), 7.49-7.51 (d, *J*= 8Hz, Ar, 1H).

^{13}C NMR ($CDCl_3$, 100 MHz) δ: 110.42, 114.41, 116.47, 119.48, 119.70, 127.00, 127.04, 128.39, 132.62, 142.45, 143.03.

HRMS (ESI): m/z [M + H^+] **calculated** for $C_{12}H_{11}BrN_2$: 263,0184**; Found:** 263.0178.

I.2– Synthesis of *N*-(2-bromophenyl)-5-methylbenzene-1,2-diamine (3b) and *N*-(2-bromophenyl)-4-methylbenzene-1,2-diamine (3c)

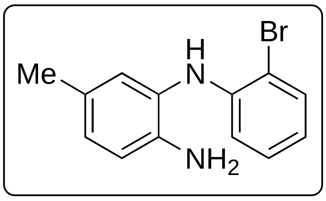

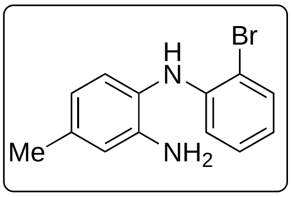

3b **3c**

Following the general Chan–Lam procedure, 4-methylbenzene-1,2-diamine (**1b**, 0.05 g, 1 equiv, 0.409 mmol) and 2-bromophenyl)boronic acid (**7**, 0.088 g, 0.409 mmol) were dissolved in dioxane (5 mL) followed by addition of CuI (0.0156 g, 0.082 mmol), Et_3N (0.068 mL, 0.49 mmol) and allowed to react as described above. After purification by silica gel chromatography (hexane/AcOEt 9:1), the compounds *N*-(2-bromophenyl)-4-methylbenzene-1,2-diamine (**3c**) and *N*-(2-bromophenyl)-5-methylbenzene-1,2-diamine (**3b**) and were obtained as a transparent oil (0.07 g, 60%). Further separation using the same eluent system allowed the separation of both compounds. Compound **3b** was eluted first to give 0.031 g, followed by 0.038 g of compound **3c**).

Data for compound 3b:

^{1}H NMR ($CDCl_3$, 400 MHz) δ: 2.27 (s, CH_3,3H), 3.54 (s, NH_2, 2H), 5.74 (s, NH, 1H), 6.61-6.63 (d, *J*= 8Hz, Ar, 1H), 6.67-6.71 (t, *J*=8Hz, Ar, 1H), 6.76-7.78 (d, *J*= 8Hz, Ar, 1H), 6.93-6.97 (m, Ar, 2H), 7.11-7.15 (t, *J*= 8Hz, Ar, 1H), 7.51-7.53 (d, *J*=8Hz, Ar,1H).

^{13}C NMR ($CDCl_3$, 100 MHz) δ: 20.46, 110.27, 114.29, 116.24, 119.51, 126.71, 127.30, 127.57,128.37, 128.62, 132.57, 140.38, 143.10.

MS (ESI) m/z: 277.10 $[M]^+$.

Data for compound 3c:
^{1}H NMR ($CDCl_3$, 400 MHz) δ: 2.30 (s, CH_3, 3H), 3.48 (s, NH_2, 2H), 5.66 (s, NH, 1H), 6.52-6.54 (d, *J*= 8Hz, Ar, 1H), 6.59-6.67 (m, Ar, 3H), 6.98-.6.70 (d, *J*= 8Hz, Ar, 1H), 7.06-7.10 (t, *J*= 8Hz, Ar, 1H), 7.46-7.49 (d, *J*= 12Hz, Ar, 1H).

^{13}C NMR ($CDCl_3$, 100 MHz) δ: 21.22, 110.03, 114.00, 116.82, 119.30, 120.08, 124.09, 127.49, 128.37, 132.52, 137.26, 142.85, 143.47.

HRMS (ESI): m/z [M + H^+] **calculated** for $C_{13}H_{13}BrN_2$: 277.0340**; Found:** 277.0332.

I.4– Synthesis of *N*^{1}-(2-bromophenyl)-4,5-dimethylbenzene-1,2-diamine (3d)

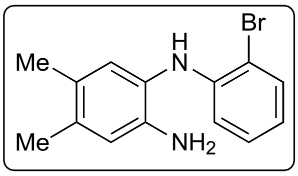

Following the general Chan–Lam procedure, 4,5-dimethylbenzene-1,2-diamine (**1c**, 0.05 g, 0.367 mmol) and (2-bromophenyl)boronic acid (**7**, 0.073 g, 0.367 mmol) were dissolved in dioxane (5 mL) followed by addition of CuI (0.014 g, 0.0734 mmol), Et_3N (0.061 mL, 0.44 mmol) and allowed to react as described above. After purification by silica gel chromatography (hexane/AcOEt, 9:1), compound **3d** was obtained as a transparent oil (0.053 g, 51% yield).

^{1}H NMR ($CDCl_3$, 400 MHz) δ: 2.15 (s, CH_3, 3H), 2.22(s, CH_3, 3H), 3.63 (s, NH_2, 2H), 5.65 (s, NH, 1H), 6.52-6.54 (d, *J*= 8Hz, Ar, 1H), 6.61-6.65 (m, Ar, 2H), 6.88 (s, Ar, 1H), 7.07-7.11 (t, *J*= 8Hz, Ar, 1H), 7.47-7.49 (d, *J*= 8Hz, Ar, 1H).
^{13}C NMR ($CDCl_3$, 100 MHz) δ: 18.79, 19.53, 109.93, 113.96, 117.61, 119.17, 124.06, 127.14, 128.38, 128.46, 132.50, 135.61, 140.99, 143.54.
HRMS (ESI): m/z [M + H^+] **calculated** for $C_{14}H_{15}BrN_2$**:** 291.0497; **Found:** 291.0491.

I.5– Synthesis of *N*^{1}-(2-bromophenyl)-4-chlorobenzene-1,2-diamine (3e)

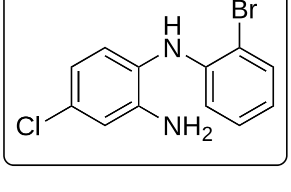

Following the general Chan–Lam procedure, 4-chlorobenzene-1,2-diamine (**1d**, 0.05 g, 0.35 mmol) and 2-(bromophenyl)boronic acid (**7**, 0.070 g, 0.35 mmol) were dissolved in dioxane (5

mL) followed by addition of CuI (20 mol %, 0.013 g, 0.07 mmol), Et_3N (0.058 mL, 1.2 equiv, 0.42 mmol) and allowed to react as described above. After purification by silica gel chromatography (hexane/AcOEt 9:1), compound **3e** was obtained as a transparent oil (0.038 g, 37% yield).

^{1}H NMR ($CDCl_3$, 400 MHz) δ: 3.90 (s, NH_2, 2H), 5.62 (s, NH, 1H), 6.52-6.54 (d, *J*= 8Hz, Ar, 1H), 6.66-6.74 (m, Ar, 2H), 6.80 (s, Ar, 1H), 7.01-7.03 (d, *J*= 8Hz, Ar, 1H), 7.09-7.13 (t, *J*= 8Hz, Ar, 1H), 7.48-7.50 (d, *J*= 8Hz, Ar, 1H).
^{13}C NMR ($CDCl_3$, 100 MHz) δ: 110.34, 114.19, 115.59, 118.79, 119.93, 125.07, 128.33, 128.44, 132.31, 132,66, 142.78, 144.38.
HRMS (ESI): m/z [M + H^+] **calculated** for $C_{12}H_{10}BrClN_2$**:** 296.9794**; Found:** 296.9788.

I.6– Synthesis of *N*^{1}-(2-bromophenyl)-4-(trifluoromethyl)benzene-1,2-diamine (3f)

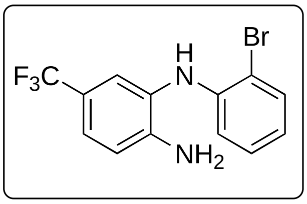

Following the general Chan–Lam procedure, 4-(trifluoromethyl)benzene-1,2-diamine (**1e**, 0.05 g, 0.28 mmol) and (2-bromophenyl)boronic acid (**7**, 0.057 g, 0.28 mmol) were dissolved in dioxane (5 mL) followed by addition of CuI (20 mol %, 0.056 mmol), Et_3N (0.047 mL, 1.2 equiv, 0.36 mmol) and allowed to react as described above. After purification by silica gel chromatography (hexane/AcOEt 9:1), compound **3f** was obtained as a transparent oil (0.026 g, 28% yield).

^{1}H NMR ($CDCl_3$, 400 MHz) δ: 4.14 (brs, NH2, 2H), 5.71 (brs, NH, 1H), 6.53-6.55 (d, *J*=8Hz, Ar, 1H), 6.70-6.73 (m, Ar, 1H), 6.83-6.85 (d, *J*=8Hz, Ar, 1H), 7.11-7.15 (t, *J*= 8Hz, Ar,1H), 7.32-7.34 (d, Ar, *J*= 8Hz,1H), 7.37 (s, Ar, 1H), 7.50-7.53 (d, *J*= 12Hz, Ar, 1H).
^{13}C NMR ($CDCl_3$, 100 MHz) δ: 110.57, 114.35, 115.25, 120.35, 124.18, 124.22, 124.25, 124.29, 126.07, 128.50, 132.75, 142.25, 146.13.
HRMS (ESI): m/z [M] $^+$ **calculated** for $C_{13}H_{10}BrF_3N_2$**:** 329.9979**; Found:** 328.9906.

I.7– Synthesis of ethyl 3-amino-4-((2-bromophenyl)amino)benzoate (3g)

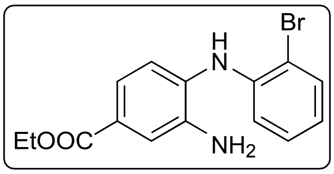

Following the general Chan–Lam procedure: ethyl 3,4-diaminobenzoate (**1f**, 0.05 g, 0.277 mmol) and (2-bromophenyl)boronic acid (**7**, 0.055g, 0.277 mmol) were dissolved in dioxane (5 mL) followed by addition of CuI (20 mol %, 0.010 g, 0.055 mmol), Et_3N (0.046 mL, 0.33 mmol) and allowed to react as described above. After purification by silica gel chromatography (hexane/AcOEt 9:1), compound **3g** was obtained as a transparent oil (0.039 g, 43% yield).

^{1}H NMR ($CDCl_3$, 400 MHz) δ: 1.33-1.37 (t, *J*=8Hz, , CH_3, 3H), 4.26 (brs.NH_2, 2H), 4.28-4.35 (m, CH_2, 2H), 5.70 (s, NH, 1H), 6.47-6.49 (d, *J*=8Hz, Ar, 1H), 6.67-6.70 (m, Ar, 1H), 6.78-6.80 (d, *J*=8Hz, Ar,1H), 7.08-7.12 (t, *J*=8Hz, Ar, 1H), 7.51-7.54 (d, *J*=8Hz, Ar, 1H), 7.80-7.82 (d, *J*=8Hz, Ar, 2H).

^{13}C NMR ($CDCl_3$, 100 MHz) δ: 14.48, 60.60, 110.74, 112.80, 121.94, 122.33, 126.89, 128.47, 129.47, 131.35, 133.12, 135.45, 136.07, 139.88, 167.49

HRMS (ESI): m/z [M + H^+] calculated for $C_{15}H_{15}BrN_2O_2$**:** 335.0340**; Found:** 335.0389.

I.8 – Synthesis of *o*-(2-bromophenylamino)-*N*-methylaniline (3h)

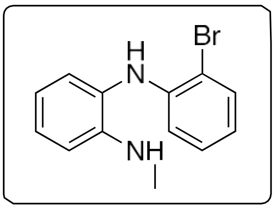

Following the general Chan–Lam procedure, *N*-methylbenzene-1,2-diamine (**1f**, 0.05 g, 0.409 mmol) and (2-bromophenyl)boronic acid (**7**, 0.082 g, 0.409 mmol) were dissolved in dioxane (5 mL) followed by addition of CuI (0.015 g, 0.082 mmol), Et_3N (0.068 mL, 0.49 mmol) and allowed to react as described above. After purification by silica gel chromatography (hexane/AcOEt 9:1), compound **3h** was obtained as a transparent oil (0.039 g, 35% yield).

^{1}H NMR ($CDCl_3$, 400 MHz) δ: 1.56 (brs, NH,1H), 3.36 (s, CH_3, 3H), 6.71 (brs, NH, 1H), 7.01-7.15 (m, Ar, 4H), 7.29-7.31 (d, *J*= 8Hz, Ar, 1H), 7.35-7.38 (m, Ar, 1H), 7.46- 7.48 (d, *J*= 8Hz, Ar,1H), 7.62-7.64 (d, *J*= 8 Hz, Ar, 1H). **^{13}C NMR ($CDCl_3$, 100 MHz) δ:** 30.1, 67.1, 108.9, 111.1, 119.36, 119.38, 126.4, 128.1, 130.6, 132.3, 135.61, 135,68, 138.2.

HRMS (ESI): m/z [M + H^+] calculated for $C_{13}H_{13}BrN_2$**:** 277.0340; **Found:** 277.0330.

II – Synthesis of dibenzodiazepinones (DBDAs)

II.1– Synthesis of 5,10-dihydro-11*H*-dibenzo[*b,e*][1,4]diazepin-11-one (4a)[1]

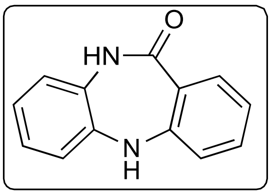

o-(2-Bromophenyl)aminoaniline (**3a**, 0.05 g, 0.19 mmol) was added to a Radley's® 12 position carousel reactor tube to which DMF, then $Pd(OAc)_2$ (4.26 mg, 0.019 mmol), DPEPhos (30 mg, 0.057 mmol), $Mo(CO)_6$ (50 mg, 1 equiv, 0.19 mmol), and Et_3N (0.026 mL, 0.19 mmol) were added. The reaction mixture was then stirred at 130 °C under a nitrogen atmosphere. After completion of the reaction, as determined by TLC, the reaction mixture was allowed to cool to room temperature. The mixture was filtered through a pad of celite and washed with DCM, then the solvent was evaporated under reduced pressure to give a crude mixture. Further purification by flash chromatography (hexane/AcOEt 1:1), gave the desired compound **4a** as a yellow solid yield (0.032 g, 80%). **M.p.:** 249-251 ºC (Lit. [1] 255-257 ºC)

^{1}H NMR (DMSO-d$_6$, 400 MHz) δ: 6.87-7.00 (m, Ar, 6H), 7.31-7.35 (t, *J*=8Hz, Ar, 1H), 7.66-7.68 (d, *J*=8Hz, Ar,1H), 7.84 (s, Ar, 1H), 9.85 (s, Ar, 1H).
^{13}C NMR (CDCl3, 100 MHz) δ:119.52, 120.23, 121.17, 121.73, 123.24, 123.40, 124.95, 130.29, 132.56, 133.67, 140.43, 150.92, 168.40.
MS (ESI) m/z: 221.12 [M+H$^+$]

II.2– Synthesis of 7-methyl-5,10-dihydro-11*H*-dibenzo[*b,e*][1,4]diazepin-11-one (4b)

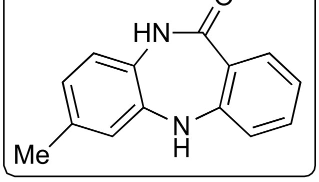

Following the general procedure, compound **3b** (0.05 g, 0.18 mmol) was dissolved in DMF, then $Pd(OAc)_2$ (4.05 mg, 0.018 mmol) and DPEPhos (29 mg, 0.054 mmol), $Mo(CO)_6$ (47 mg, 0.18 mmol), and Et_3N (0.026 mL, 0.19 mmol) were added to a Radley's® 12 position carousel reactor tube. Purification with flash chromatography using (hexane/AcOEt 1:1), as eluent gave the desired compound **4c** as a yellow solid yield (0.038 g, 95%). **M.p.:** 205-210 ºC. (Lit. [2]; 255-257 ºC)

^{1}H NMR (DMSO-d$_6$, 400 MHz) δ: 1.33 (s, CH_3, 3H), 5.86-5-88 (d, *J*=8Hz, Ar,1H), 5-96-6.05(m, Ar, 3H), 6-12-6.14 (d, *J*=8Hz, Ar, 1H), 6.46-6.50 (t, *J*= 8Hz, Ar, 1H), 6.80-6.82 (d, *J*= 8Hz, Ar, 1H), 6.90 (s, Ar, 1H), 8.91 (s, Ar, 1H)
^{13}C NMR (DMSO-d$_6$, 100 MHz) δ: 20.71, 119.48, 120.61, 121.12, 121.64, 123.31, 123.90, 127.75, 132.52, 133.53, 134.08, 140.33, 150.89, 168.32.
MS (ESI) m/z: 225.15 [M+H$^+$]

II.3 – Synthesis of 8-methyl-5,10-dihydro-11*H*-dibenzo[*b*,*e*][1,4] diazepin-11-one (4c)[1].

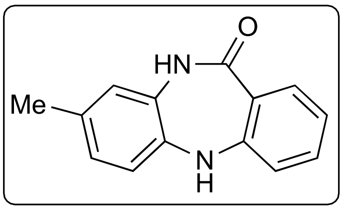

Following the general procedure, compound **3c** (0.05 g, 0.18 mmol) was dissolved in DMF, then $Pd(OAc)_2$ (4.05 mg, 0.018 mmol), DPEPhos (29 mg, 0.054 mmol), $Mo(CO)_6$ (47 mg, 0.18 mmol), and Et_3N (0.026 mL, 0.19 mmol) were added to a Radley's® 12 position carousel reactor tube. Purification with flash chromatography using (hexane/AcOEt 1:1), as eluent, gave the desired compound **4b** as a yellow solid yield (0.037 g, 93%). **M.p.:** 197-200 ºC. (Lit.[1], 200-204ºC)

^{1}H NMR (DMSO-d$_6$, 400 MHz) δ: 2.14 (s, CH_3, 3H), 6.72-6.74 (m, Ar, 2H), 6.83-6.86 (m Ar, 2H), 6.93-6.95(d, *J*=8Hz, Ar, 1H), 7.27-7-31(t, *J*= 8Hz, Ar, 1H), 7.62-7.64 (dd, *J*= 8Hz, Ar, 1H), 7.70 (s, Ar,1 H), 9.75 (s, Ar, 1H).
^{13}C NMR (DMSO-d6, 100 MHz) δ: 20.64, 119.37, 120.13, 120.99, 122.00, 123.19, 125.41, 130.11, 132.37, 132.54, 133.57, 137.91, 151.19, 168.49.
MS (ESI) m/z: 225.15

II.4 – Synthesis of 7,8-dimethyl-5,10-dihydro-11*H*-dibenzo[*b*,*e*][1,4]diazepin- 11-one (4d)

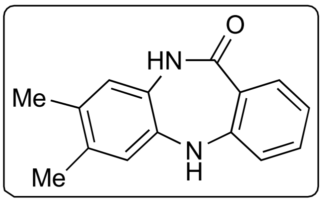

Following the general procedure, compound **3d** (0.05 g, 0.17 mmol) was dissolved in DMF, then $Pd(OAc)_2$ (3.86 mg, 0.017 mmol) and DPEPhos (28 mg, 0.052 mmol), $Mo(CO)_6$ (44 mg, 0.17 mmol), and Et_3N (0.023 mL, 0.17 mmol) were added to a Radley's® 12 position carousel reactor tube. Purification with flash chromatography using (hexane/AcOEt 1:1), as eluent gave the desired compound **4d** as a yellow solid yield (0.03 g, 75%). **M.p.:** 209-215 ºC. (Lit. [3], off white solid).

^{1}H NMR (DMSO-d$_6$, 400 MHz) δ: 1.22-1.24 (d, *J*=8Hz, 6H), 5.86 (s, Ar, 1H), 5.91(s, Ar, 1H), 5.99-6.03 (t, *J*= 8Hz, Ar, 1H), 6.09-6.11(d, *J*= 8Hz, Ar,1H), 6.43-6.45(t, *J*= 8Hz, Ar, 1H), 6.79-6.80 (d, *J*=4Hz, Ar, 2H), 8.84(s, Ar, 1H).
^{13}C NMR (DMSO-d$_6$, 100 MHz) δ:31.17, 119.34, 120.92, 121.23, 122.62, 123.26, 127.70, 130.87, 132.53, 133.45, 137.99, 151.25, 168.46, 207.04.
HRMS (ESI): m/z [M+H$^+$] **calculated** for $C_{16}H_{14}N_2O_3$**:** 239.1184**; Found** 239,1175.

II.5 – Synthesis of 8-chloro-5,10-dihydro-11*H*-dibenzo[*b*,*e*][1,4]diazepin-11-one (4e) [1]

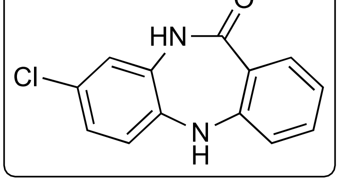

Following the general procedure, compound **3e** (0.05 g, 1 equiv, 0.168 mmol) was dissolved in DMF, then $Pd(OAc)_2$ (3.77 mg, 10 mol %, 0.0168 mmol) and DPEPhos (27 mg, 30 mol %, 0.050 mmol), $Mo(CO)_6$ (44 mg, 1 equiv,0.168 mmol), and Et_3N (0.023 mL, 1 equiv, 0.17 mmol) were added to a Radley's® 12 position carousel reactor tube. Purification with flash chromatography using (hexane/AcOEt 1:1) as eluent gave the desired compound **4e** as a yellow solid yield (0.035 g, 85%). **M.p.:** 235-237 ºC.

^{1}H NMR (DMSO-d$_6$, 400 MHz) δ: 6.03-6.07 (t, *J*=8Hz, Ar, 1H), 6.11-6.14 (m, Ar, 4H), 6.48-6.52 (t, *J*=8Hz, Ar, 1H), 6.81-6.83 (d, *J*=8Hz, Ar, 1H), 7.13 (s, Ar, 1H), 9.07 (s, Ar, 1H). **^{13}C NMR (DMSO-d$_6$, 100 MHz) δ:** 119.56, 120.90, 121.43, 121.51, 122.82, 124.46, 126.71, 131.66, 132.66, 133.99, 139.19, 150.25, 168.12.
HRMS (ESI): m/z [M + H$^+$] **calculated** for $C_{16}H_{14}N_2O_3$**:** 245.0482; **Found** 245.0475.

II.6 – Synthesis of 7-(trifluoromethyl)-5,10-dihydro-11*H*-dibenzo[*b*,*e*][1,4]diazepin-11-one (4f)[1]

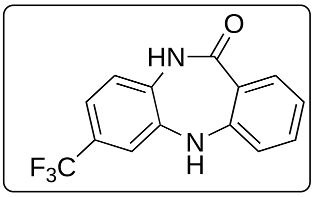

Following the general procedure, compound **3f** (0.05 g, 0.15 mmol) was dissolved in DMF, then Pd(OAc)$_2$ (3.36 mg, 0.015 mmol) and DPEPhos (24 mg, 0.045 mmol), Mo(CO)$_6$ (39 mg, 0.15 mmol), and Et$_3$N (0.020 mL, 0.17 mmol) were added to a Radley's® 12 position carousel reactor tube. Purification with flash chromatography using (hexane/AcOEt 1:1), gave the desired compound **4f** as a yellow solid yield (0.023 g, 55%). **M.p.:** 200-205 ºC. (Lit. [1], 202-203 ºC)

^{1}H NMR (DMSO-d$_6$, 400 MHz) δ: 6.91-6.95 (t, *J*=8Hz, Ar, 1H), 6.97-6.99 (d, *J*=8Hz, Ar, 1H), 7.11-7.13 (d, *J*= 8Hz, Ar, 1H), 7.24-7.26 (d, *J*=8Hz, Ar, 1H), 7.35-7.40 (m, Ar, 2H), 7.69-7.71(d, *J*= 8 Hz, Ar, 1H), 8.14 (s, Ar, 1H), 10.15 (s, Ar, 1H).**^{13}C NMR (DMSO-d$_6$, 100 MHz) δ:** 116.82, 119.64, 120.17, 121.70, 122.05, 122.78, 123.41, 126.44, 132.72, 133.93, 134.20, 140.28, 149.71, 167.95.
HRMS (ESI): m/z [M + H$^+$] **calculated** for $C_{14}H_9F_3N_2O$**:** 279.0745; **Found:** 279.0734.

II.7– Synthesis of ethyl 11-oxo-10,11-dihydro-5*H*-dibenzo[*b*,*e*][1,4]diazepine-8-carboxylate (4g)

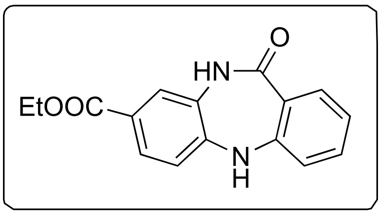

Following the general procedure, compound **3g** (0.05 g, 0.15 mmol) was dissolved in DMF, then $Pd(OAc)_2$ (3.36 mg, 0.015 mmol) and DPEPhos (24 mg, 0.045 mmol), $Mo(CO)_6$ (39 mg, 0.15 mmol), and Et_3N (0.020 mL, 0.17 mmol) were added to a Radley's® 12 position carousel reactor tube. Purification with flash chromatography using (hexane/AcOEt 1:1), gave the desired compound **4g** as a yellow solid yield (0.016 g, 40%). **M.p.:** 225-230 ºC.

^{1}H NMR (DMSO-d$_6$, 400 MHz) δ: 1.29-1.31 (t, *J*= 4Hz, CH_3), 4.23-4.29 (q, *J*=8Hz, CH_2),6.91-6.94(m, Ar, 1H), 6.99-7.01 (d, *J*=8Hz, Ar,1H), 7.05-7.07 (d, *J*= 8Hz, Ar, 1H), 7.35-7.39(t, *J*= 8Hz, Ar, 1H), 7.55-7.59 (t, *J*= 8Hz, Ar, 2H), 7.70-7.72 (d, *J*= 8Hz, Ar, 1H), 8.34 (s, Ar, 1H), 9.97 (s, Ar, 1H).
^{13}C NMR (DMSO-d$_6$, 100 MHz) δ: 14.68, 31.18, 60.93, 119.70, 119.99, 121.61, 122.58,122.67, 124.50, 126.26, 129.80, 132.80, 134.07, 144.51, 149.08, 165.56, 167.75.
HRMS (ESI): m/z [M + H$^+$] **calculated** for C16H14N2O3**:** 283.1083; **Found** 283.1073.

III – Kinetic study of the Molybdenum/Palladium Buchwald–Hartwig coupling reaction

In order to understand the role of the Molybdenum species in this reaction, and if it exerted a catalytic effect, a kinetic study was performed using the aforementioned model system described above with 5 mol % of $Mo(CO)_6$. The standard reaction (benchmark) contained everything bar the Mo species. A 1 mL sample was collected every 10 min and the reaction progress was constantly monitored by ^{1}H NMR (Scheme S1). For practical reasons, the reaction was carried out at a larger scale than in the previous reactions, and the results are shown in Figure S1. At the larger scale, we observed the formation of the usual black precipitate in the presence of $Mo(CO)_6$ which could be due to the complexation of the palladium with the molybdenum, which may lead to a reduction in the catalytic activity of that particular system.

Palladium catalysts have a tendency for aggregation which often leads to the formation of a black precipitate and we believe this phenomenon can also take place at the larger scale used here [4]. The $Mo(CO)_6$ may also exert a reducing effect on the Pd(II) catalyst, producing Pd(0) as black precipitate. For practical reasons, the reaction was only monitored for 90 min. In all cases, except for the first 10 min, the reaction without Mo gave the best yields, this was particularly evident after 30 min.

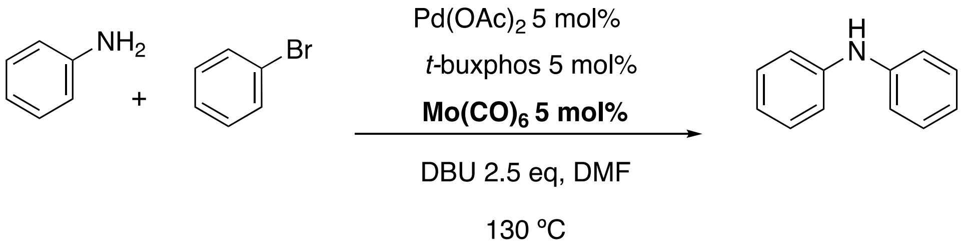

Scheme S1: Mo-catalyzed Buchwald–Hartwig coupling of bromobenzene with aniline.

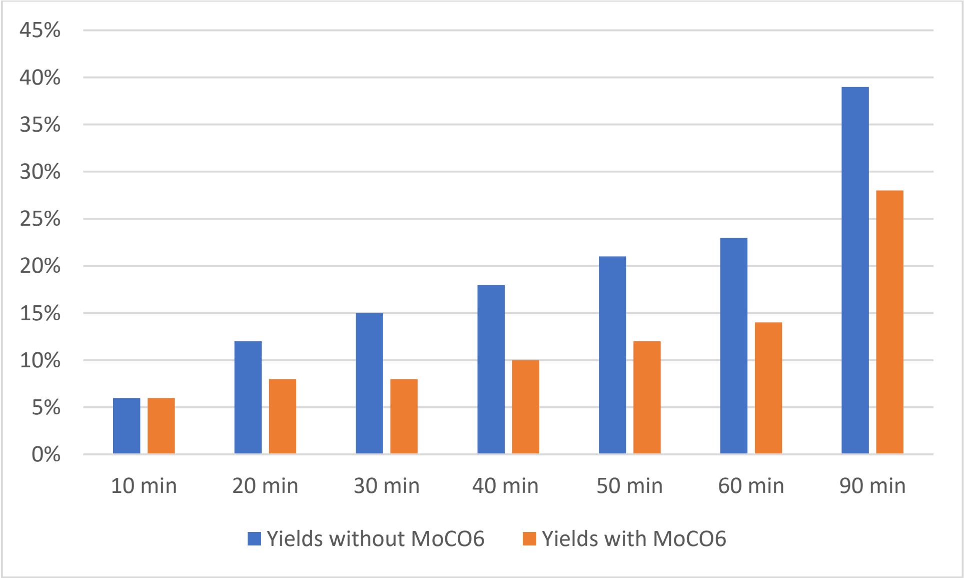

Figure S1: Comparative study of the Pd and Pd/Mo-catalyzed B-H amination between bromobenzene and aniline (analysis conducted via ^{1}H NMR with a mesitylene standard).

In a round-bottomed flask, aniline (0.45 mL, 5 mmol), *o*-bromobenzene (0.52 mL, 5 mmol) were dissolved in DMF (20 ml), then $Pd(OAc)_2$ (56 mg, 0.25 mmol), DPEPhos (0.13 g, 0.25 mmol) and $Mo(CO)_6$ (66 mg, 0.25 mmol) were added to the reaction mixture. The resulting mixture was allowed to stir at 130 °C under a nitrogen atmosphere. A sample (1 mL) was taken from the reaction mixture after 5 min, 10 min, 20 min, 30 min, 40 min, 50 min, 60 min, and 90

min of reaction. The sample was filtered through a glass pipette with flash silica and washed with DCM. The solvent was evaporated under reduced pressure, and mesitylene (0.08 g, 0.48 mmol) was added as standard to each crude mixture and analyzed by ^{1}H NMR, the yields were determined afterwards.

Table S1: Kinetic study of the Pd/Mo catalyzed B-H coupling of aniline with bromobenzene.

Time (min)	Without $MoCO_6$ (%)	With $MoCO_6$ (%)
10	6	6
20	12	8
30	15	8
40	18	10
50	21	12
60	23	14
90	39	28

IV NMR spectra of *o*-(2-bromophenyl)aminoanilines

IV.1– NMR data for 3a

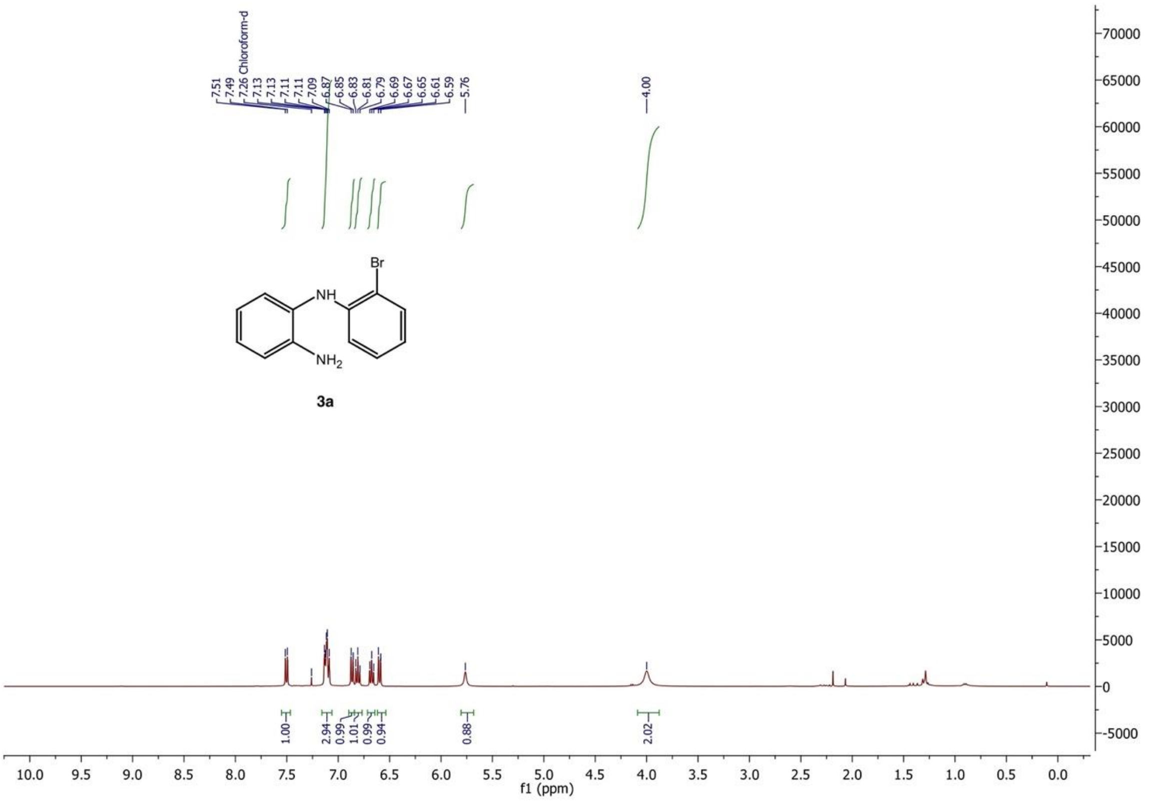

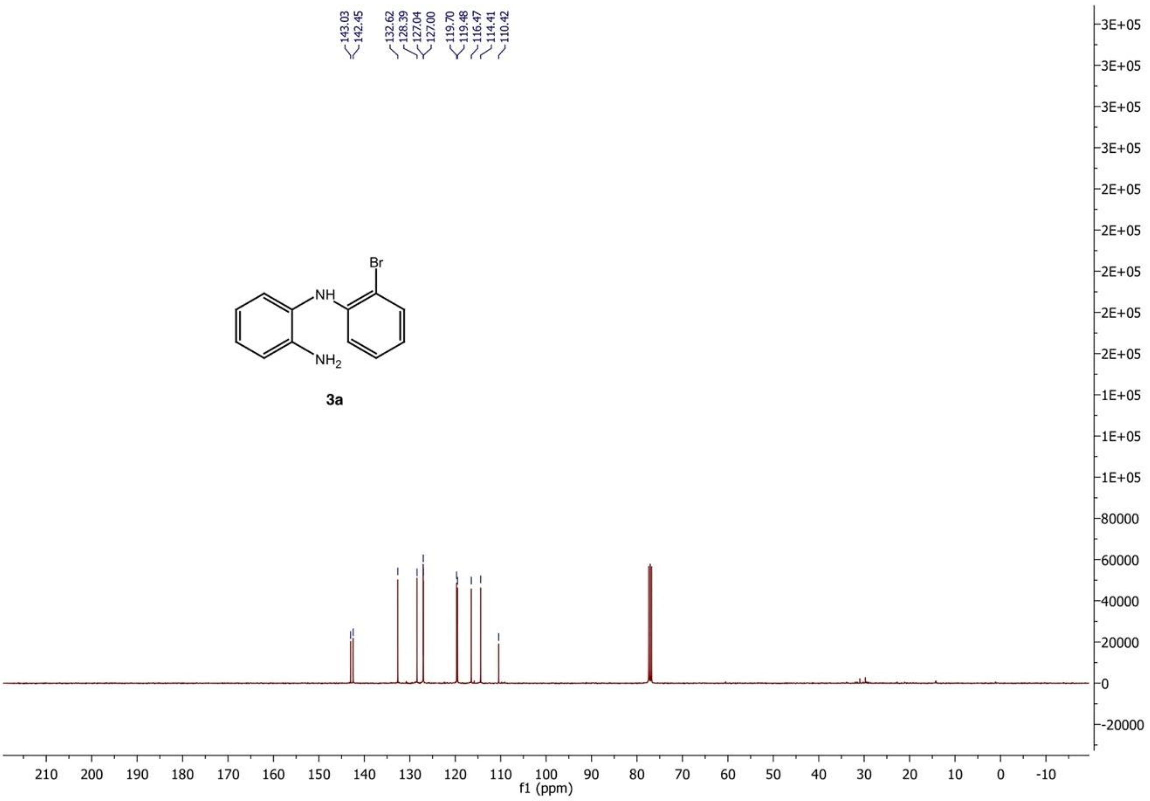

IV.2– NMR data for compound 3b

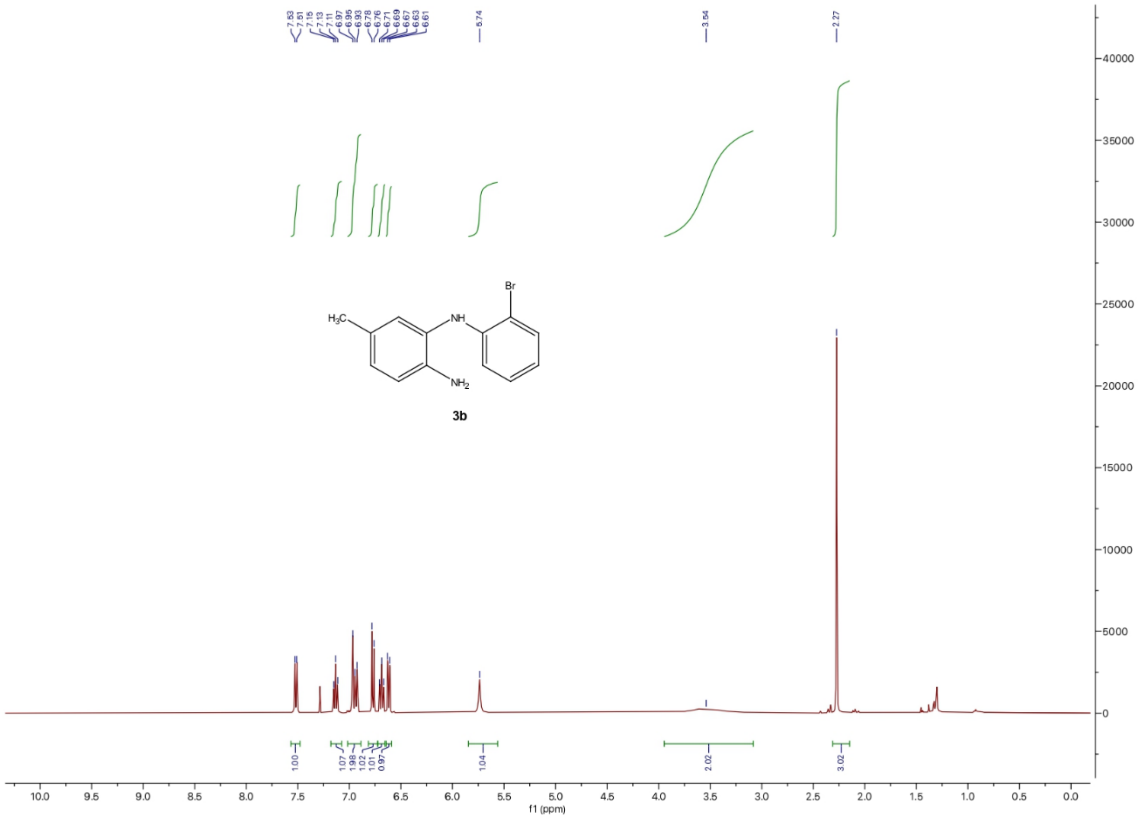

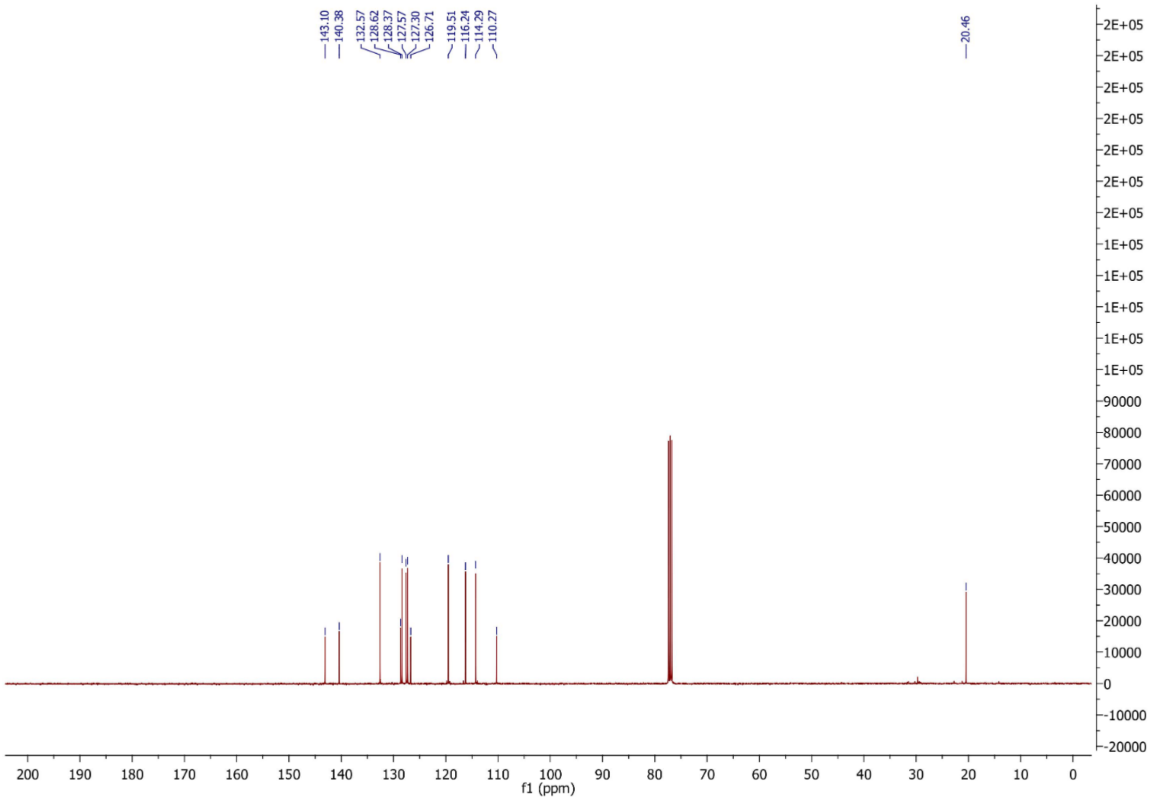

IV.3– NMR data for compound 3c

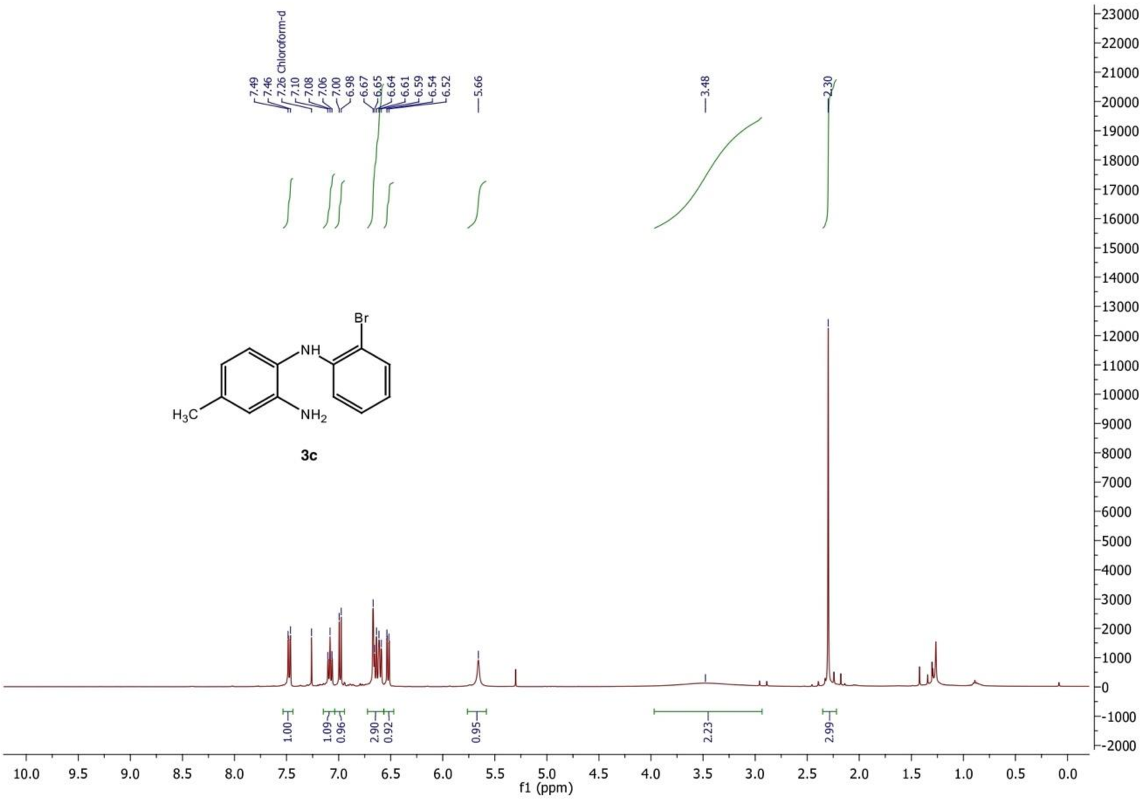

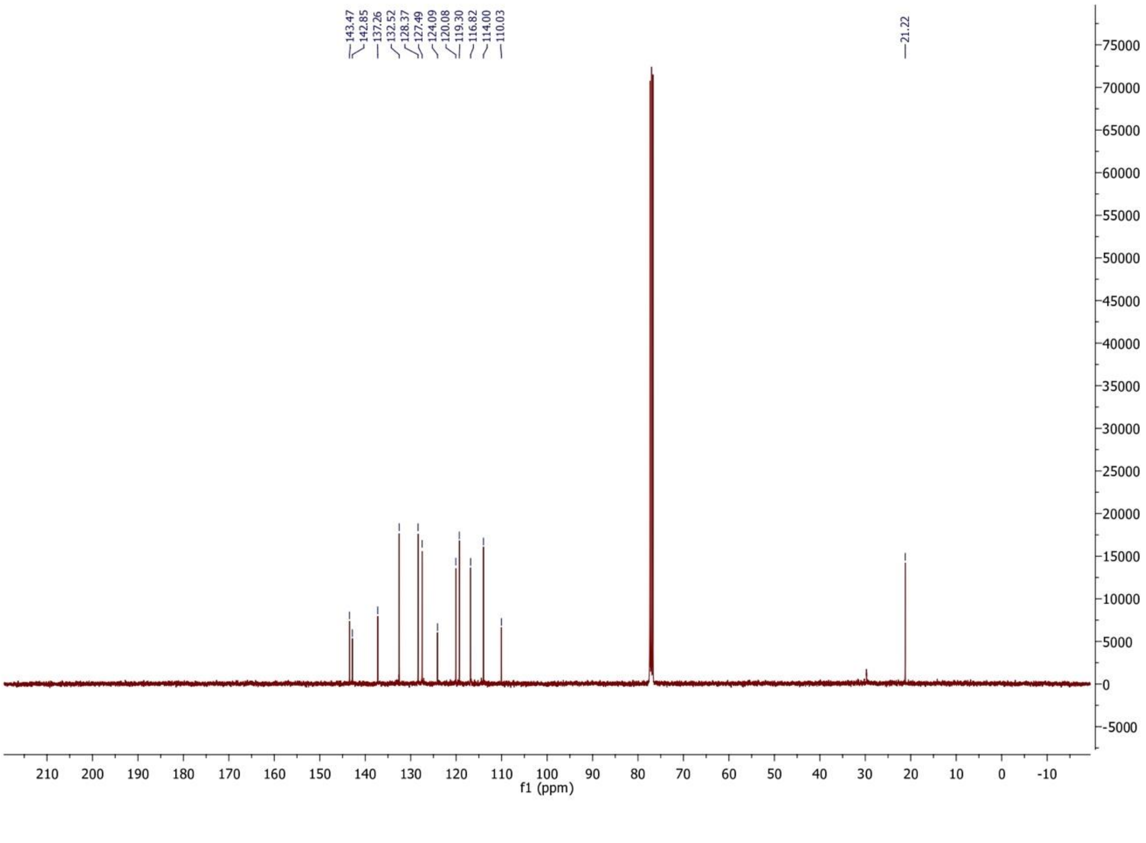

IV.4– NMR data forcompound 3d

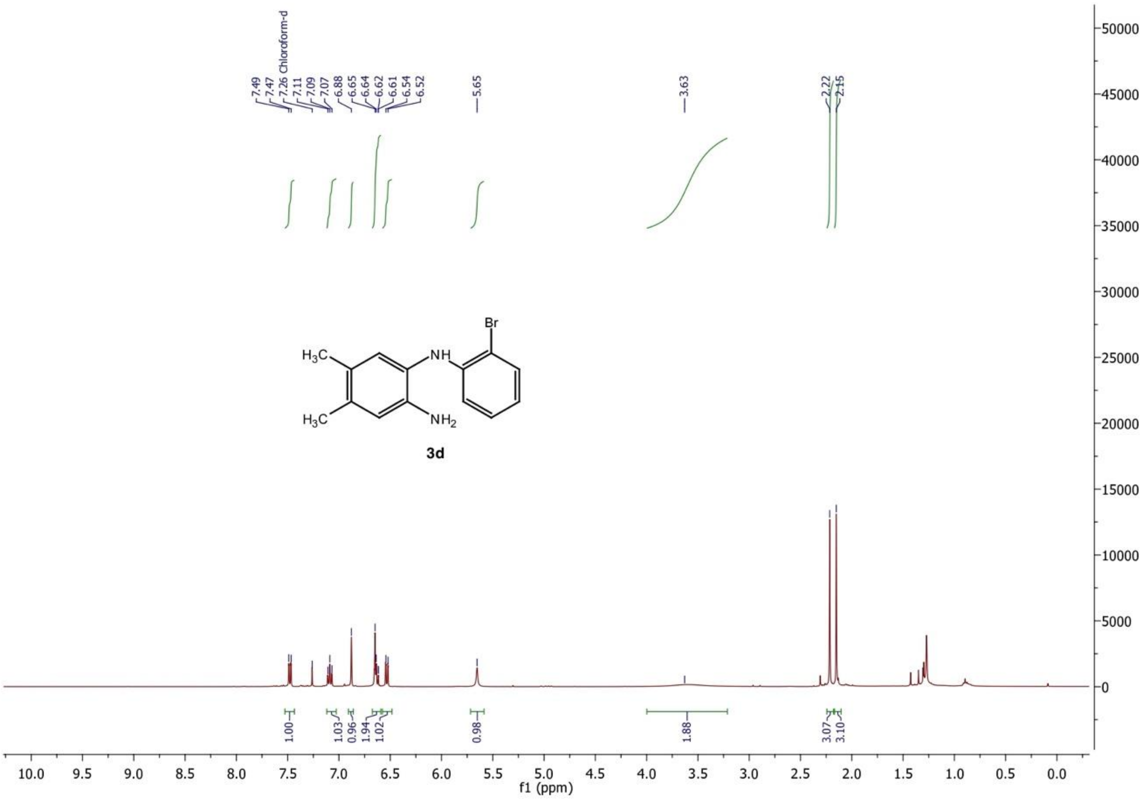

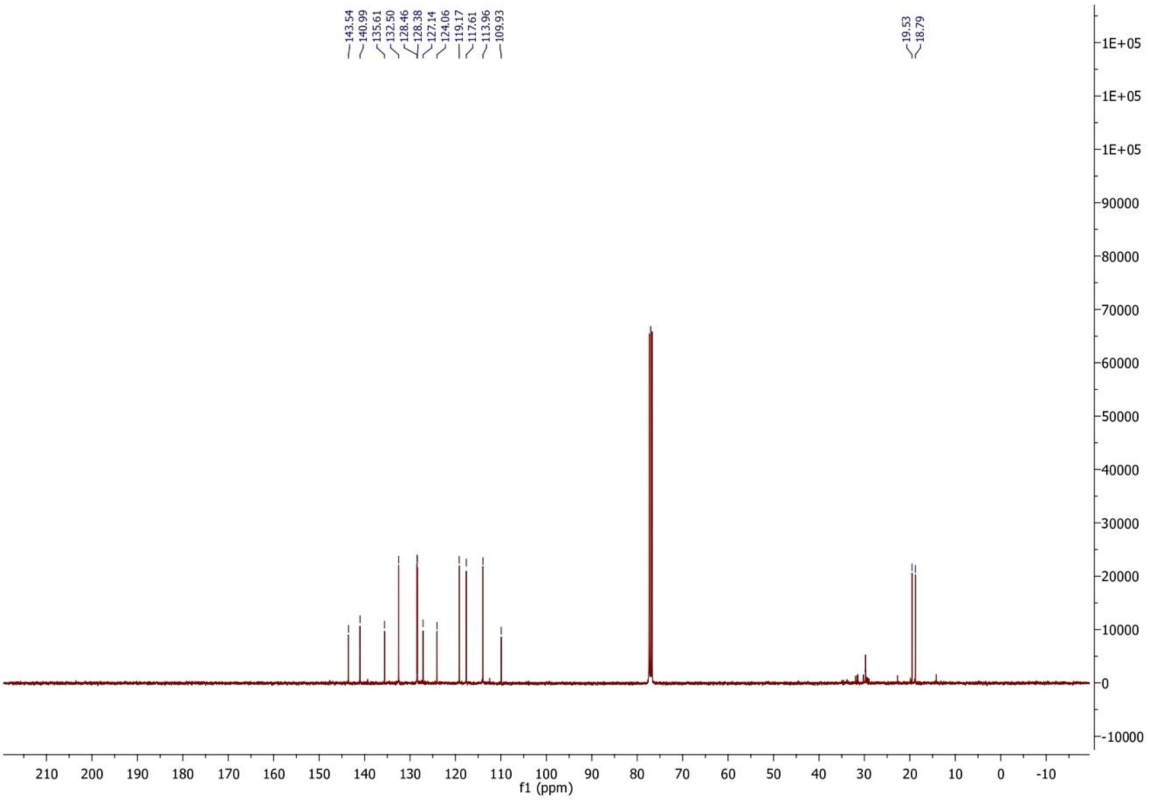

IV.5– NMR data for compound 3e

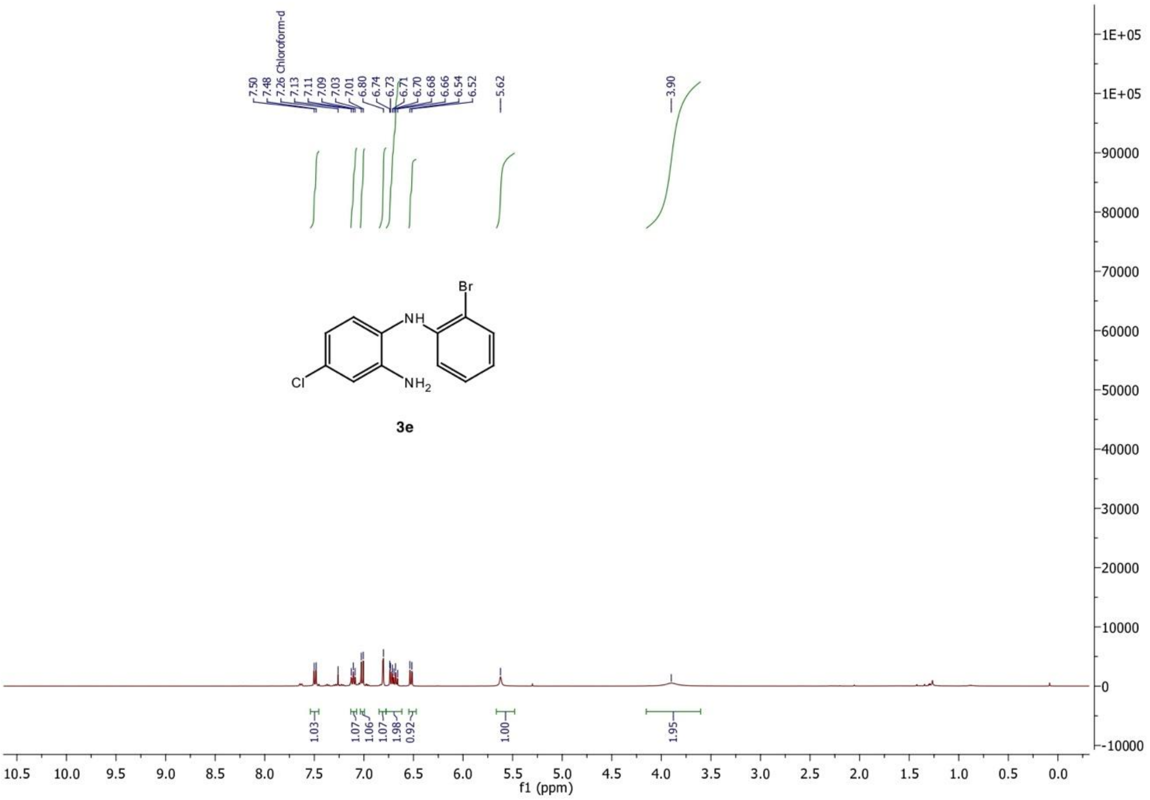

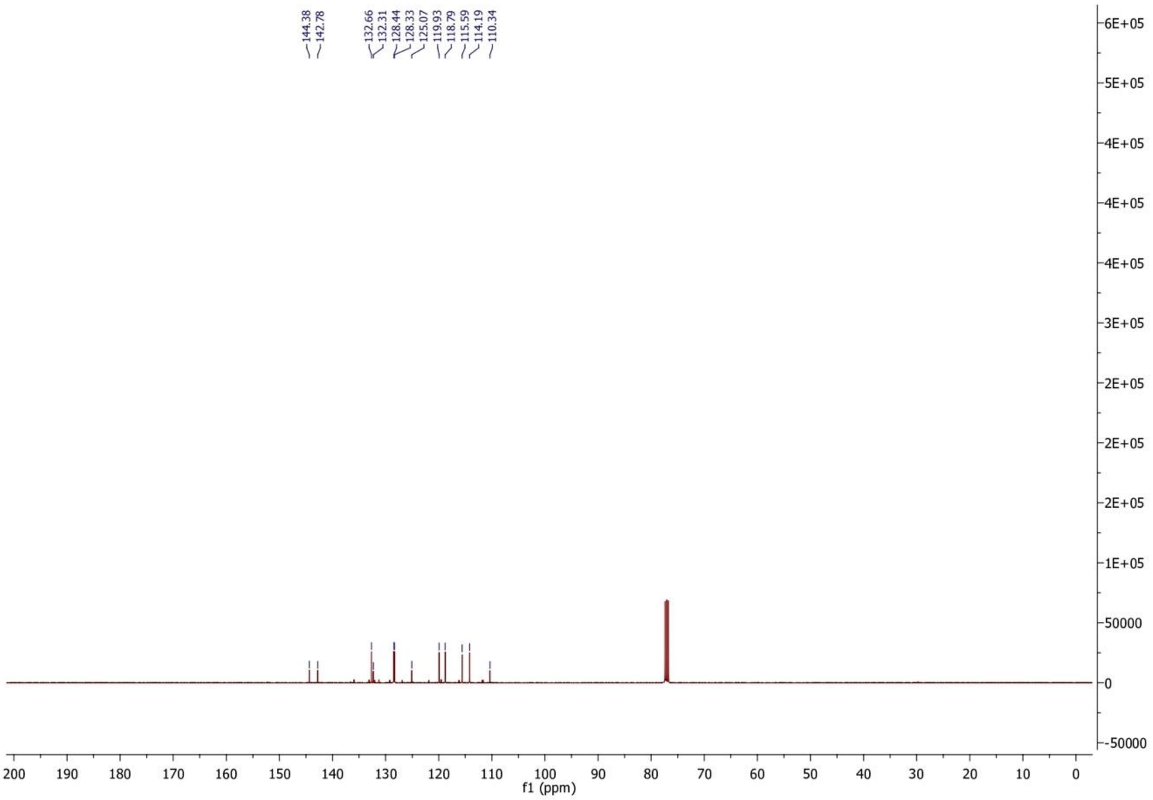

IV.6– NMR data for compound 3f

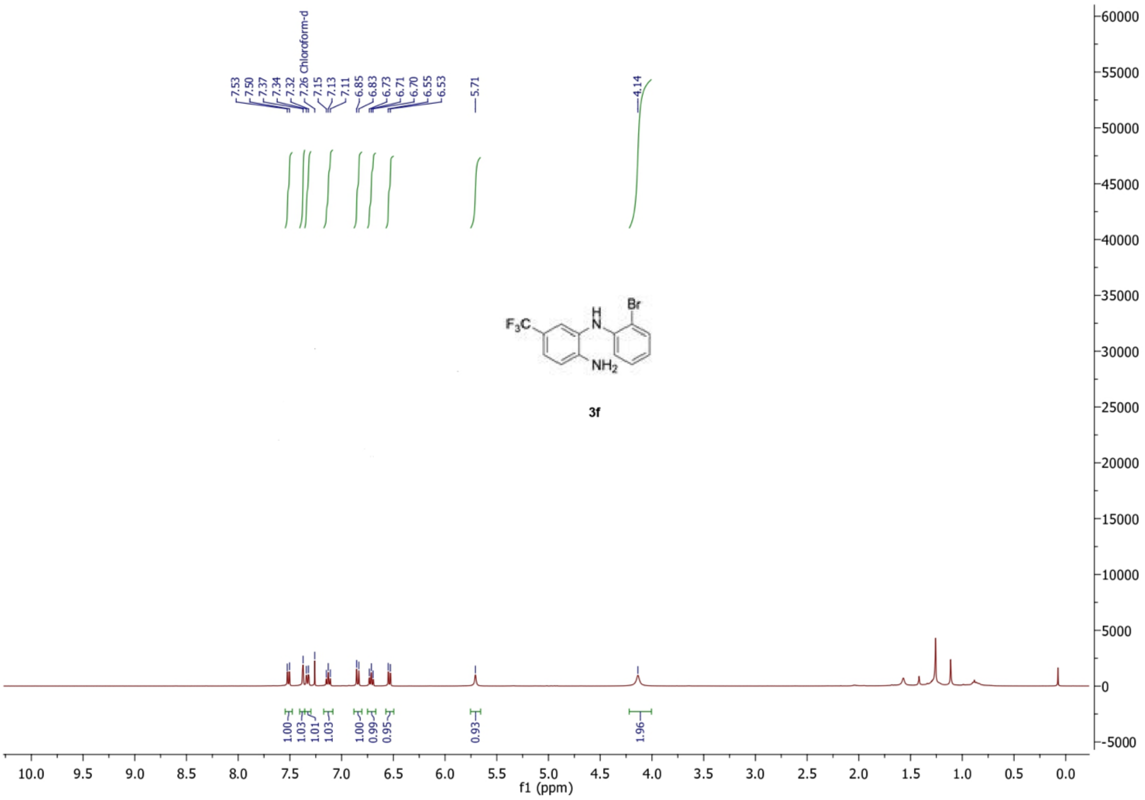

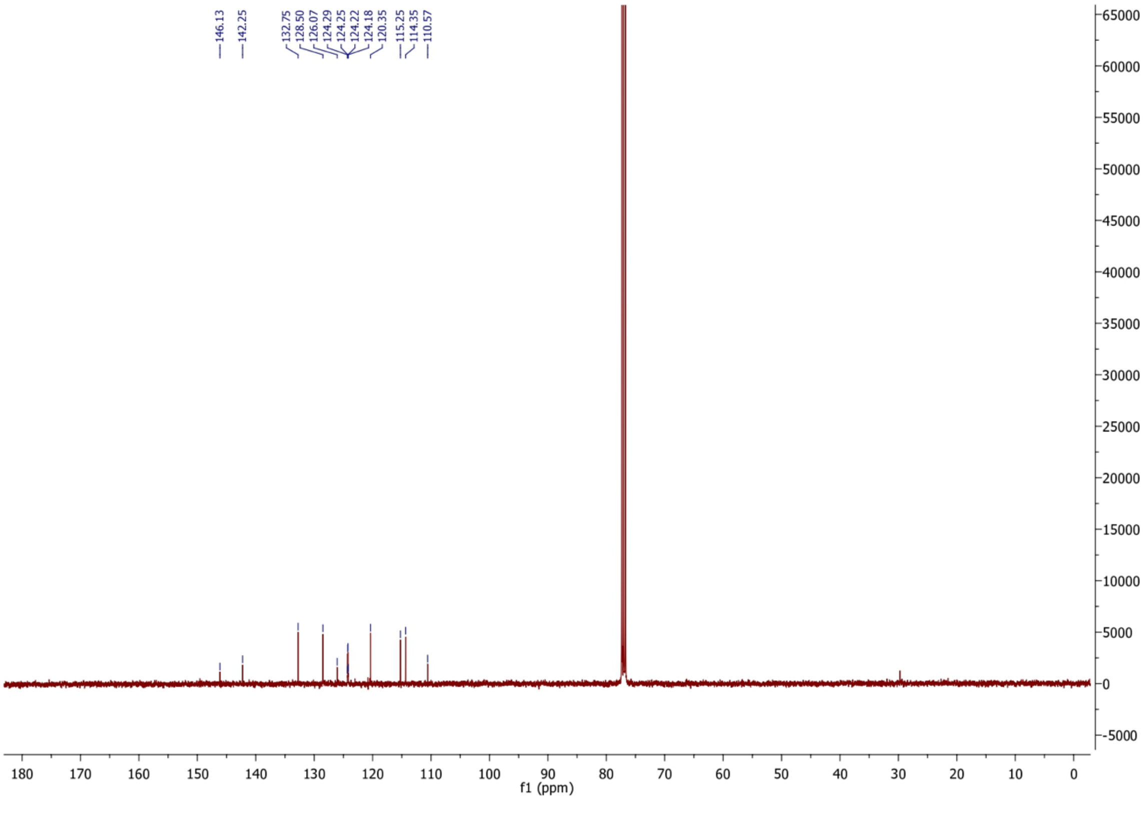

IV.7– NMR data for compound 3g

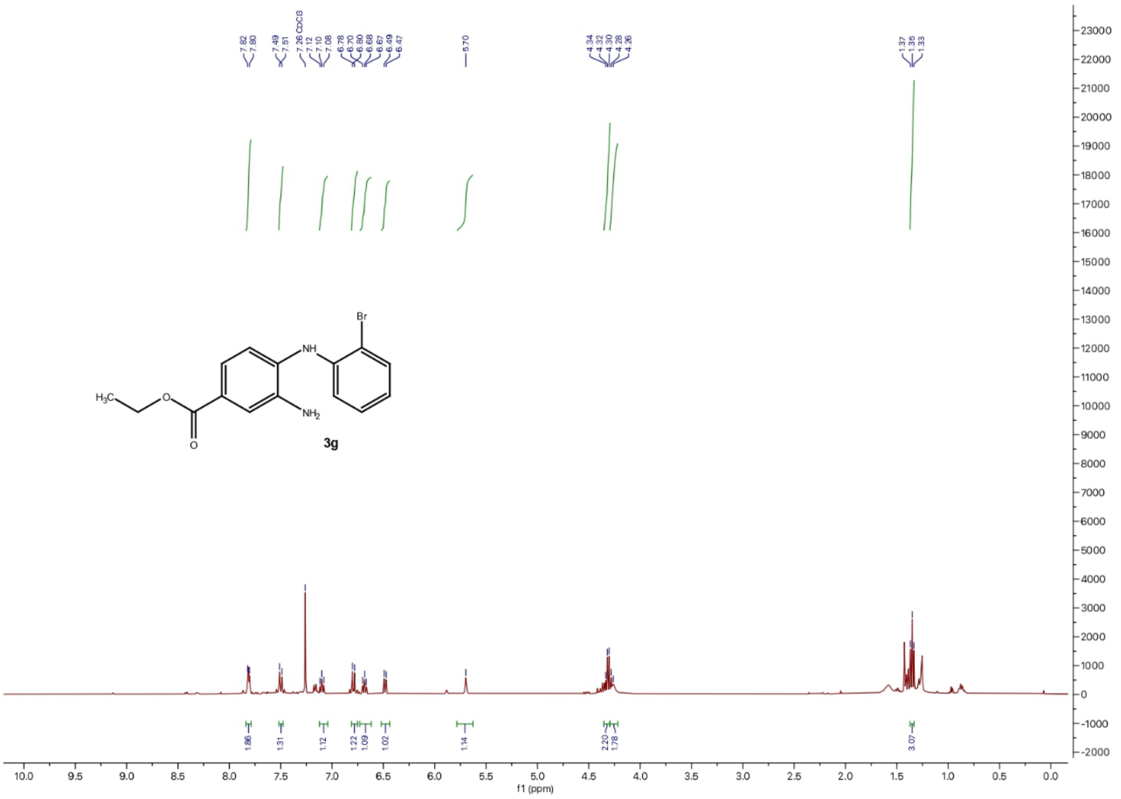

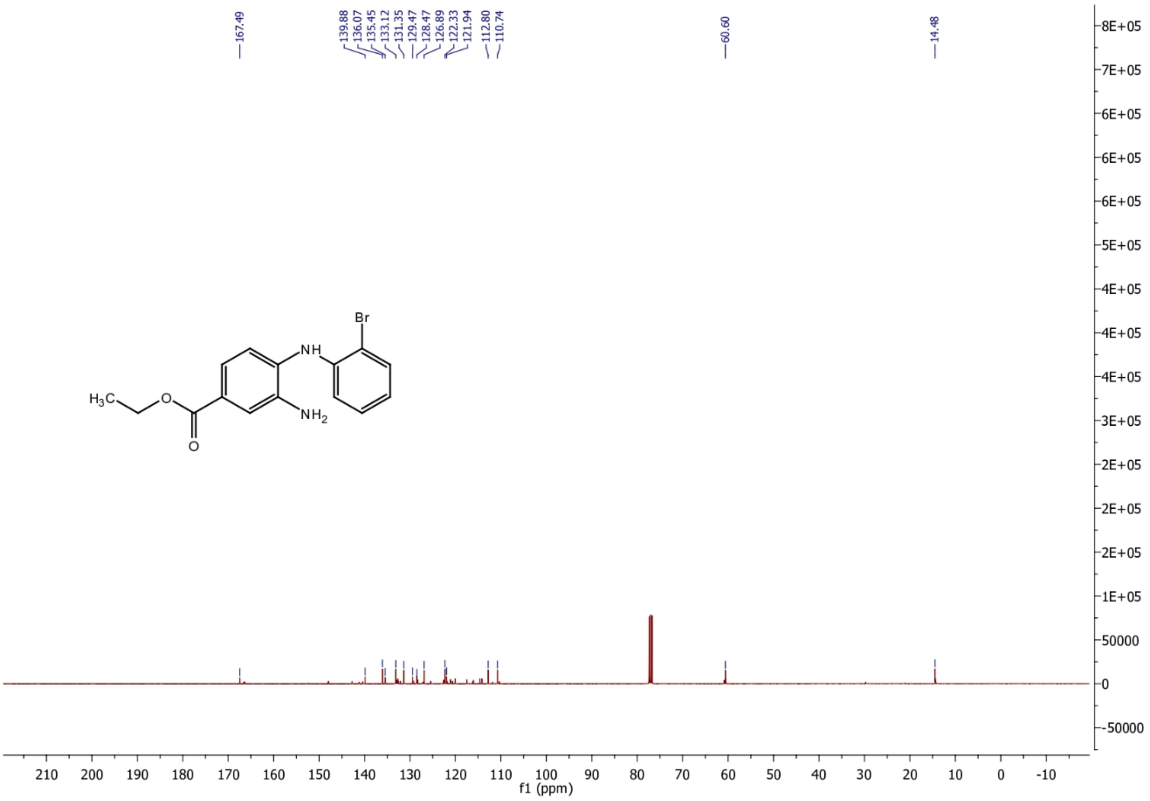

IV.7– ^{1}H NMR spectrum for compound 3h

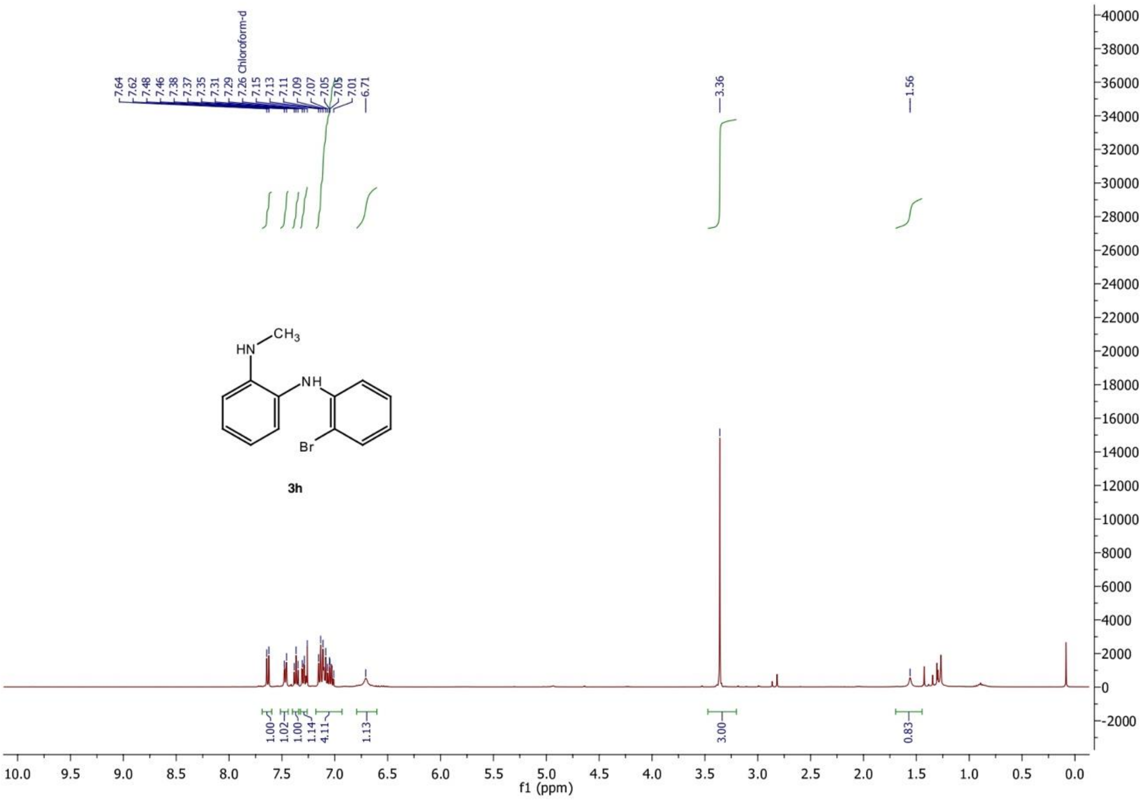

IV.8– Mass spectrum of compound 3h

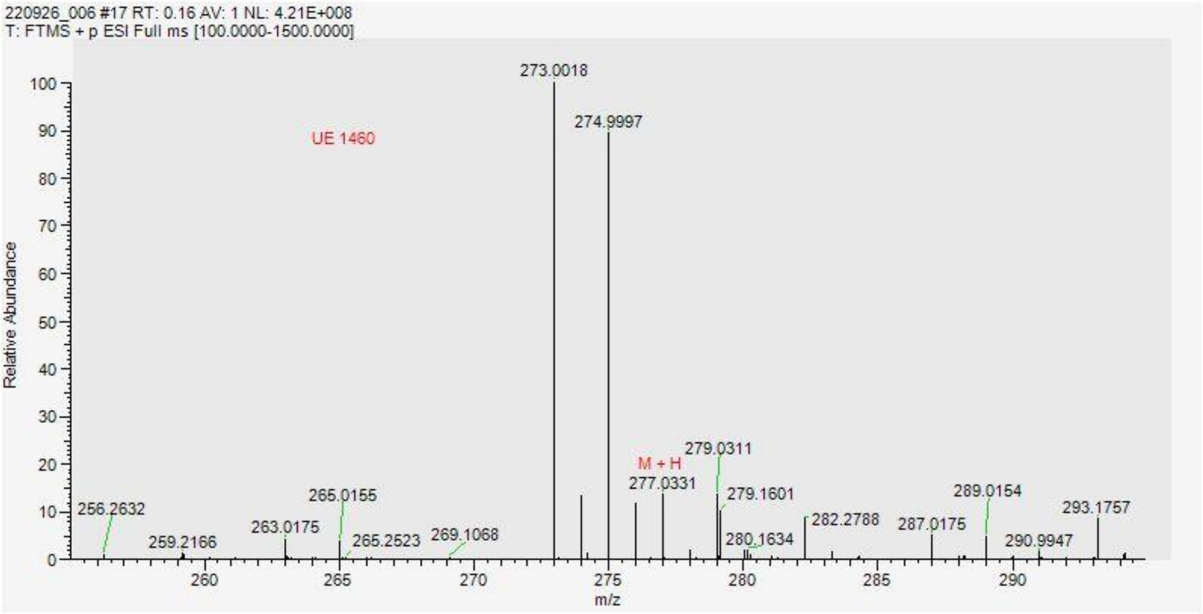

V– NMR spectra for 5,10-dihydro-11*H*-dibenzo[*b*,*e*][1,4]diazepin-11-ones

V.1– NMR data for compound 4a

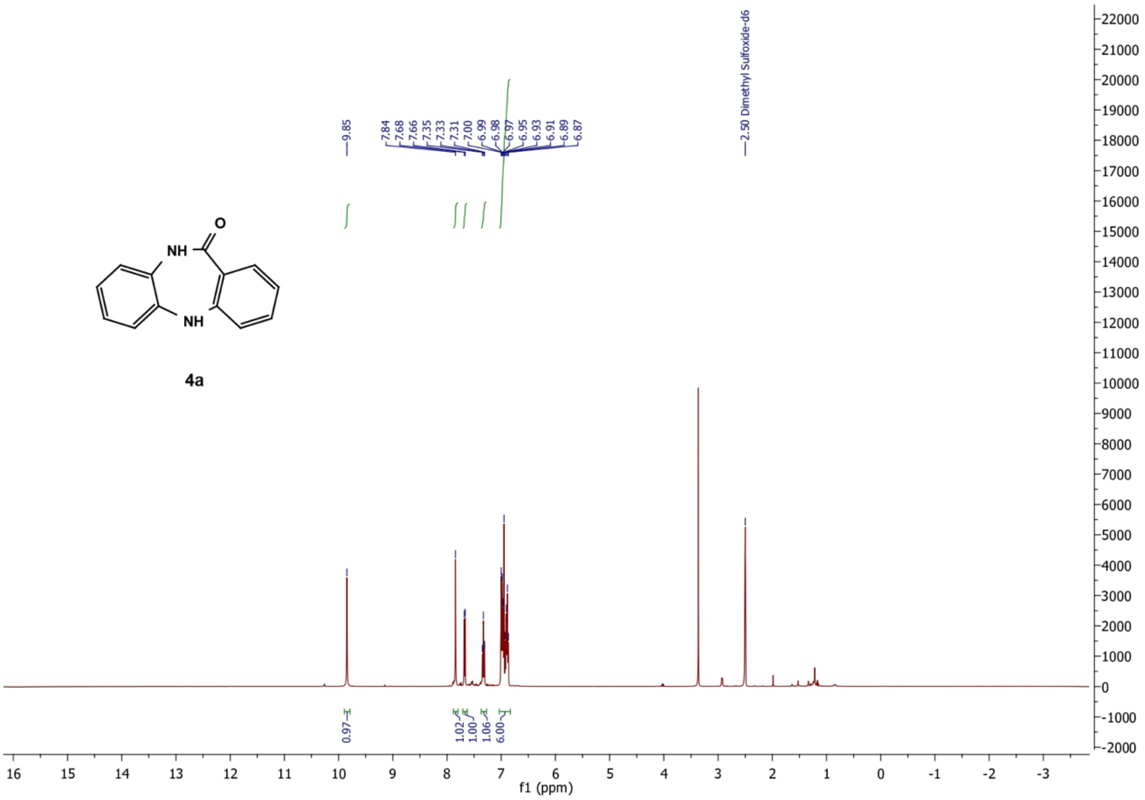

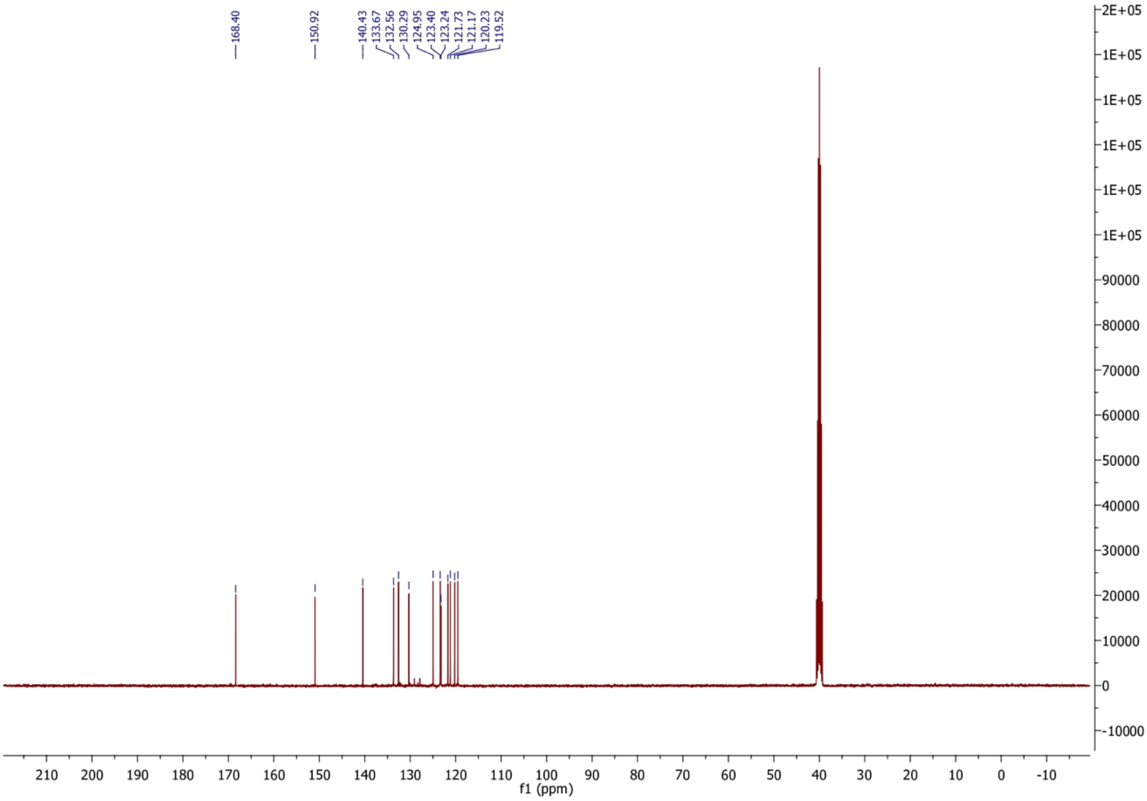

V.2– NMR data forcompound 4b

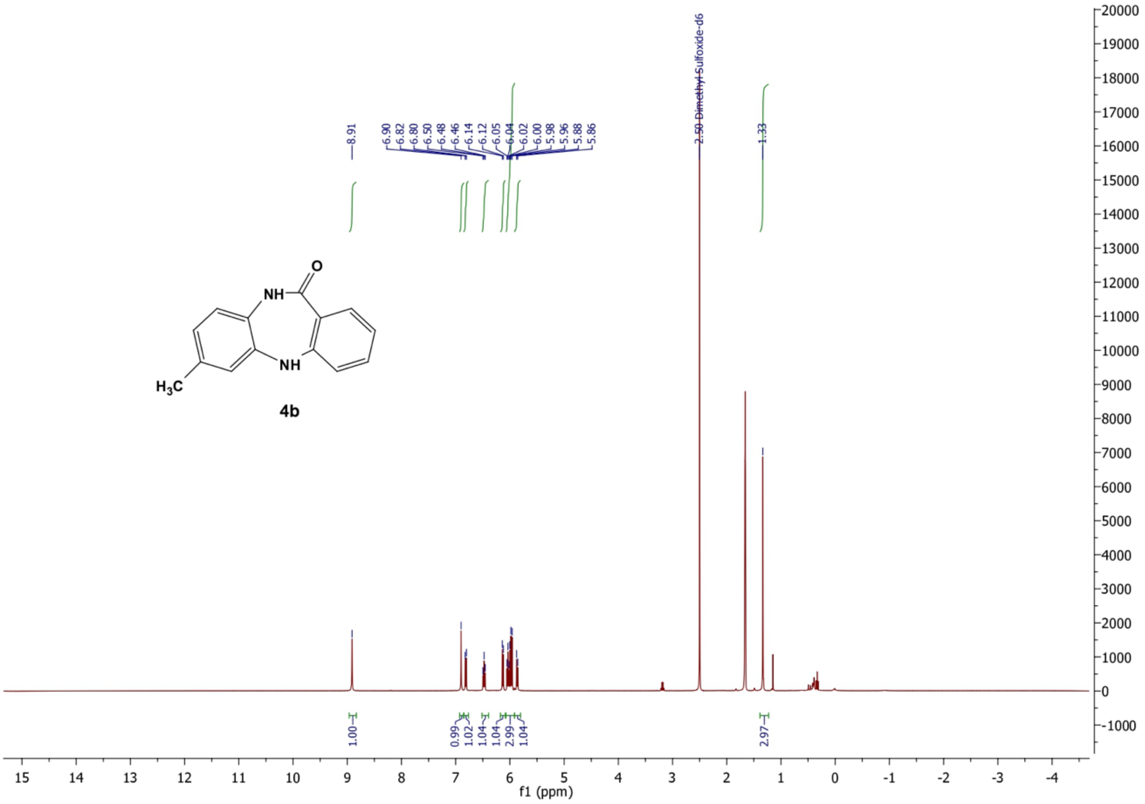

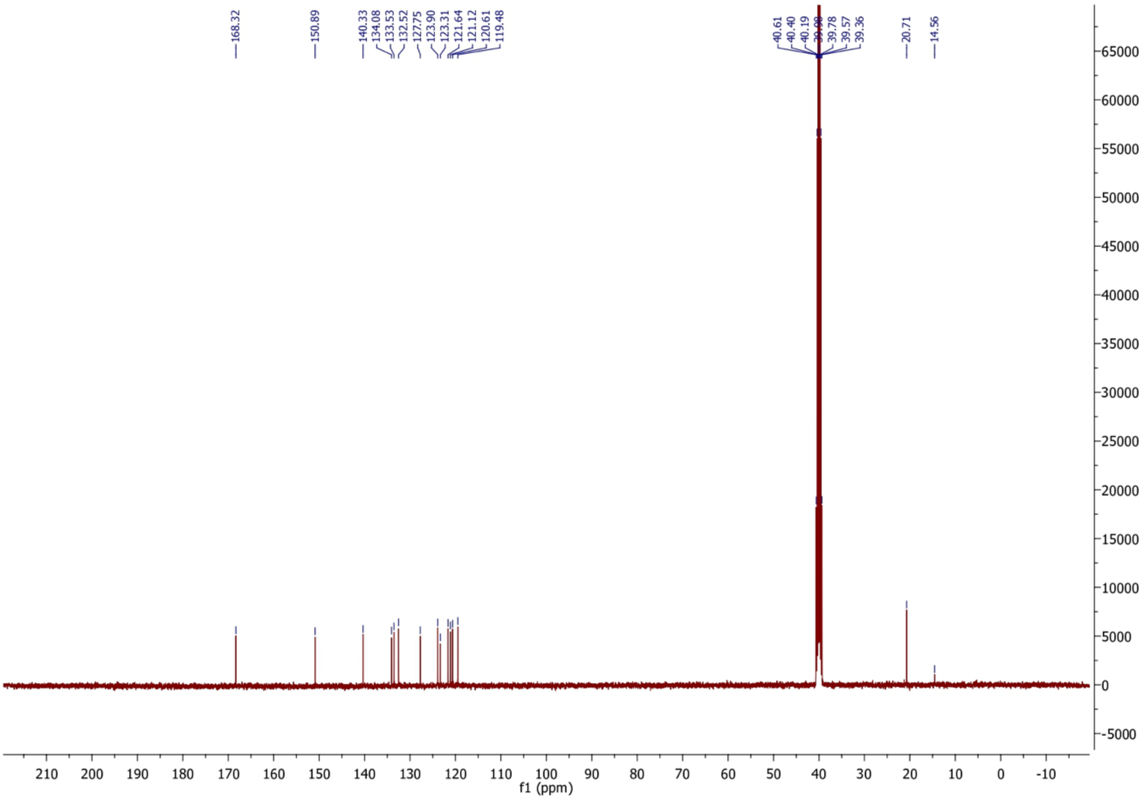

V.3– NMR data for compound 4c

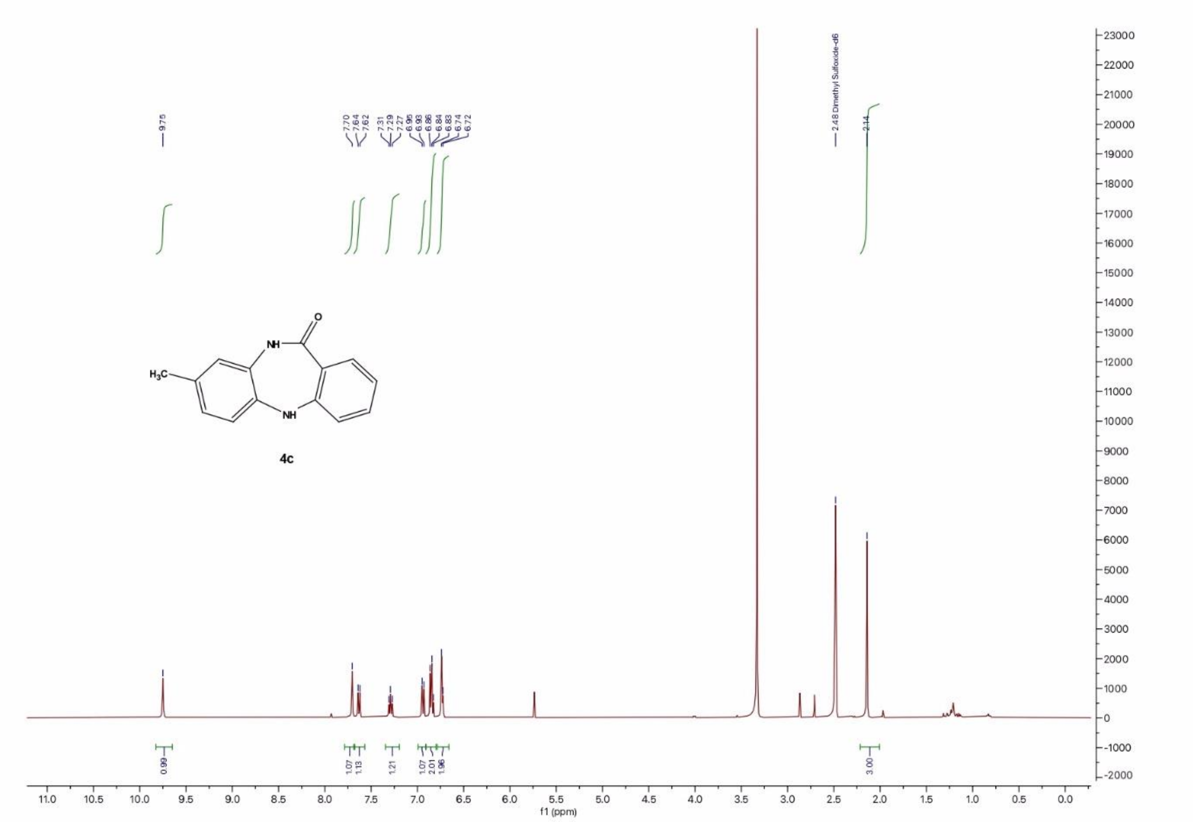

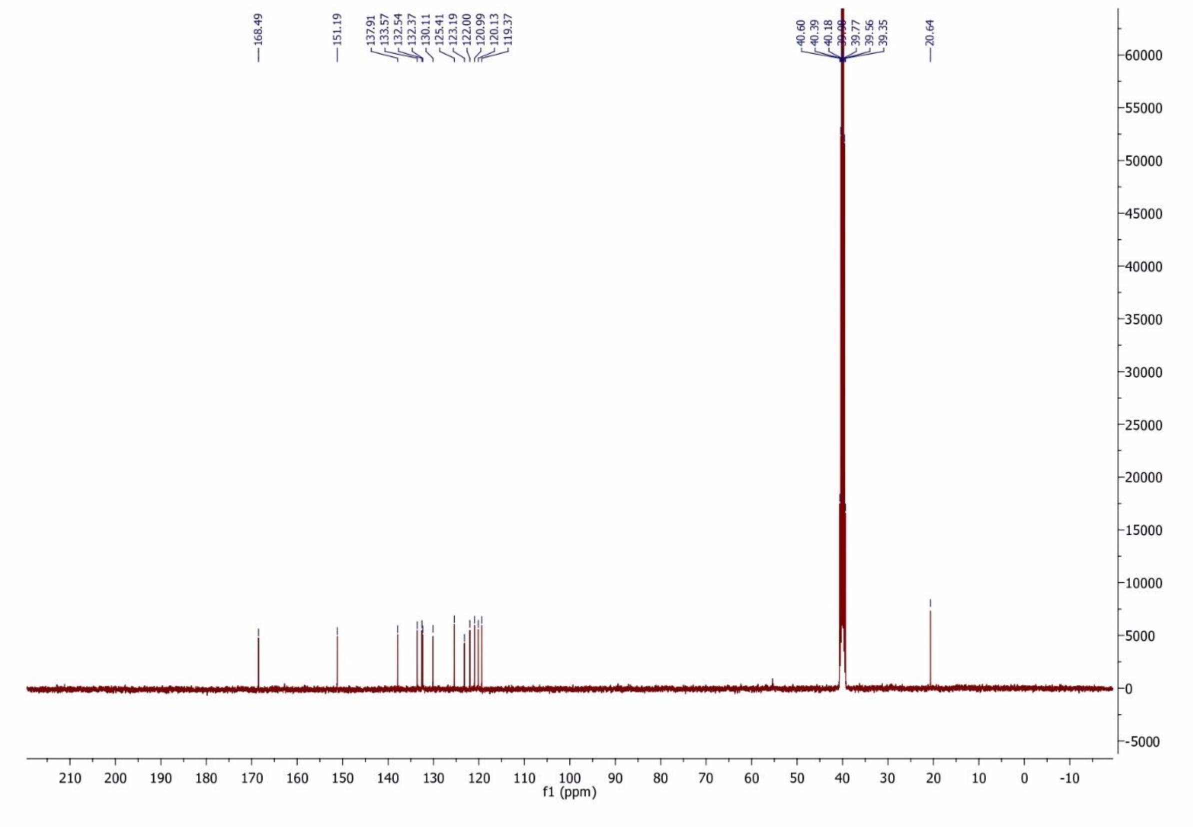

V.4– NMR data for compound 4d

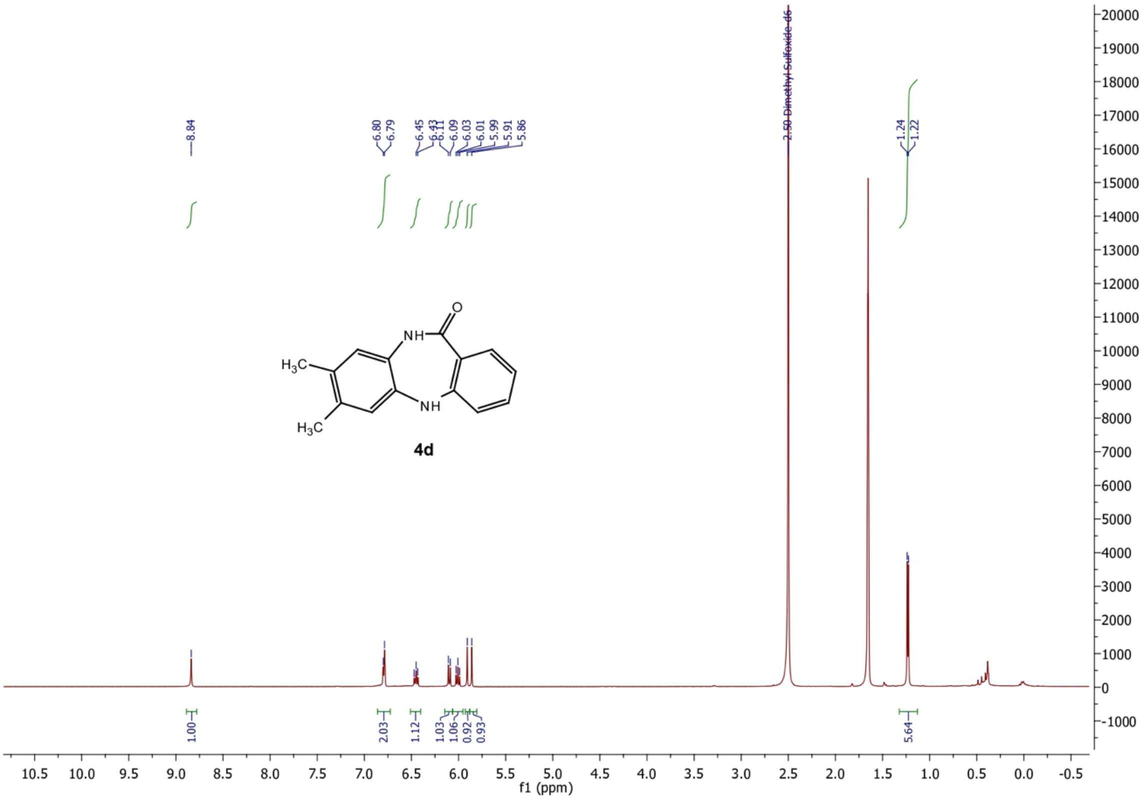

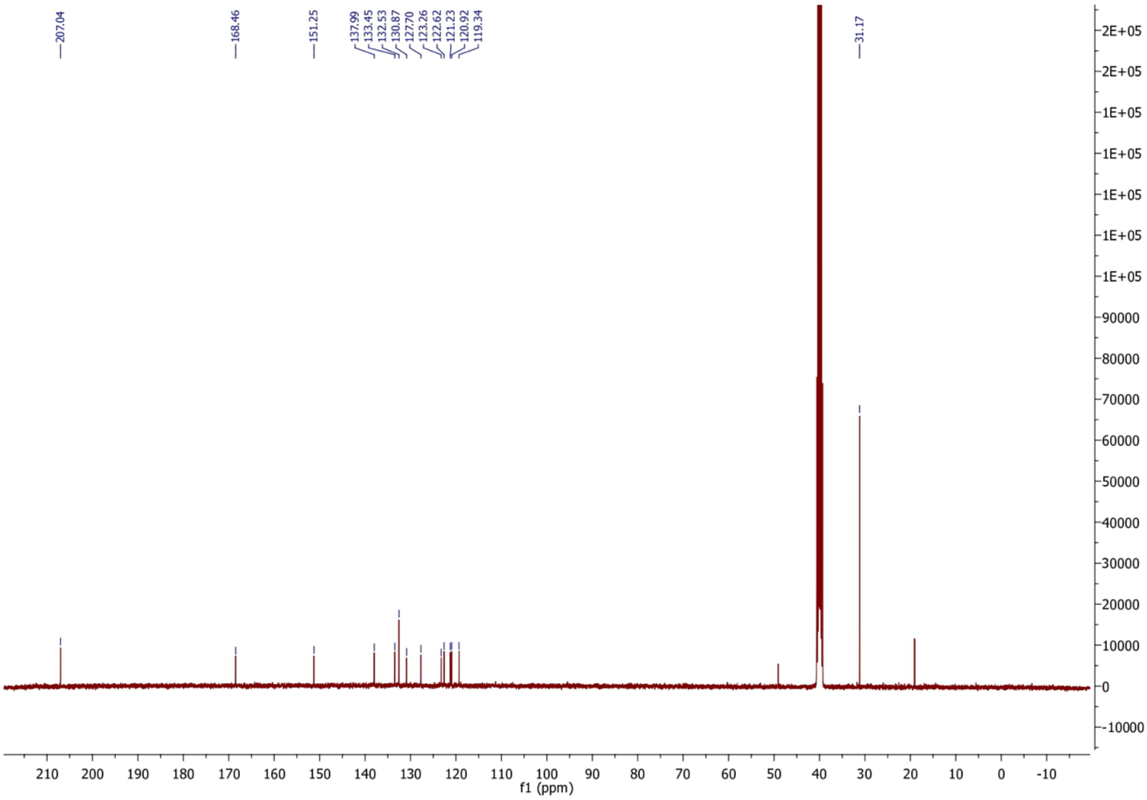

V.5– NMR data for compound 4d

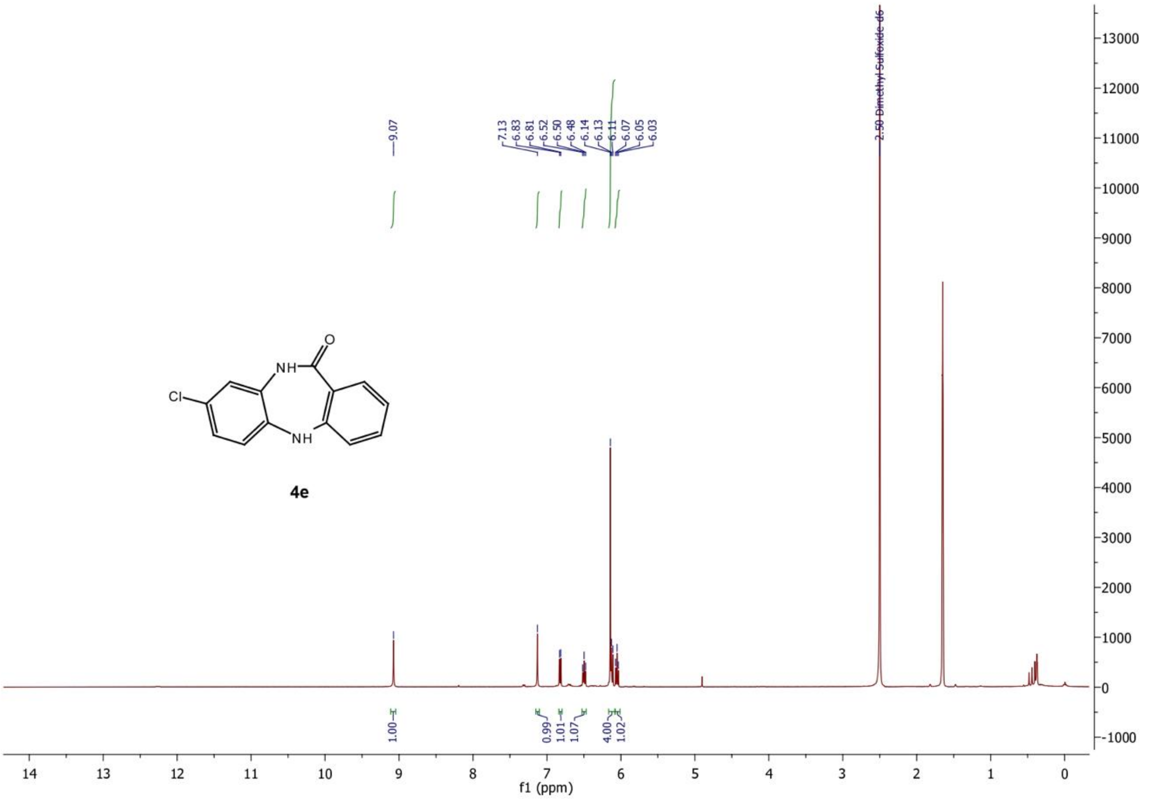

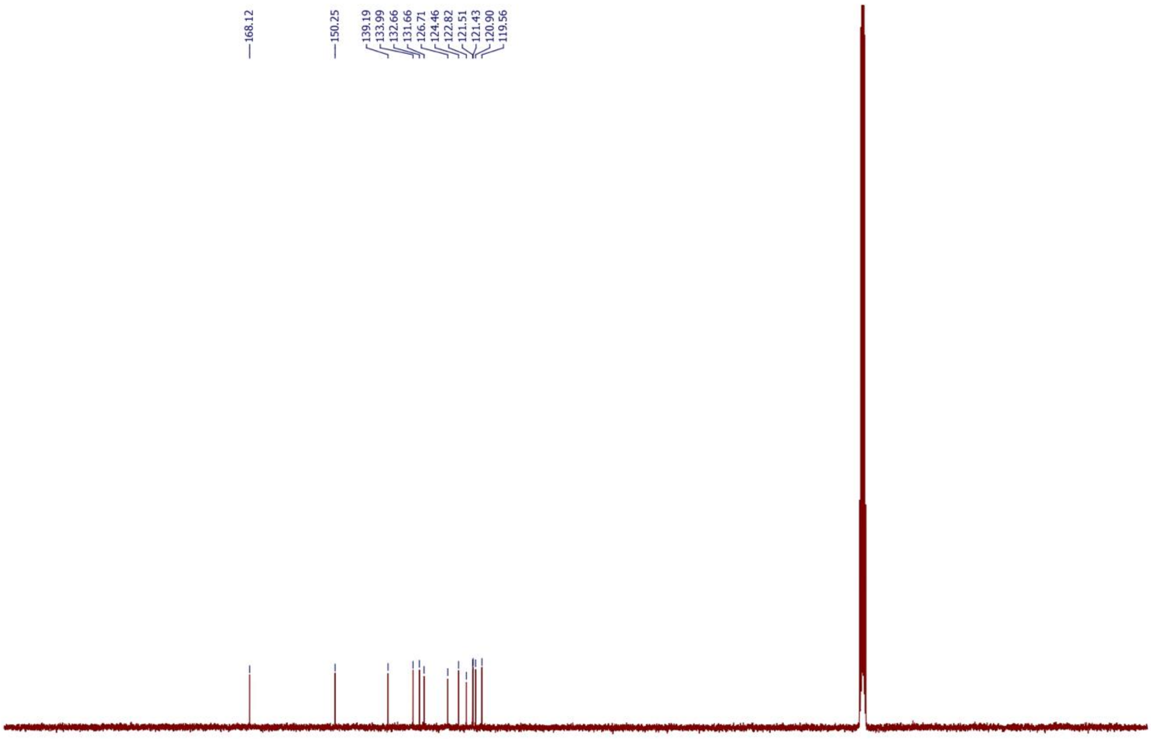

V.6– NMR data for compound 4f

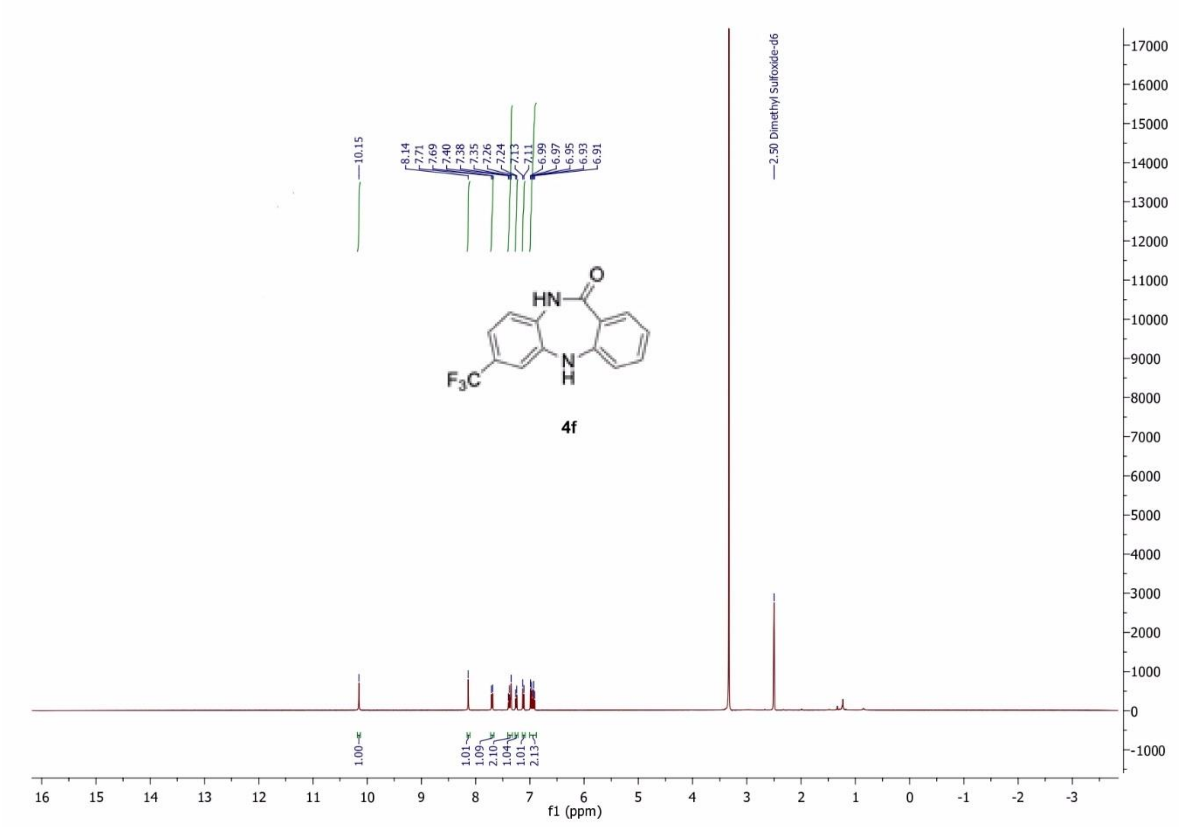

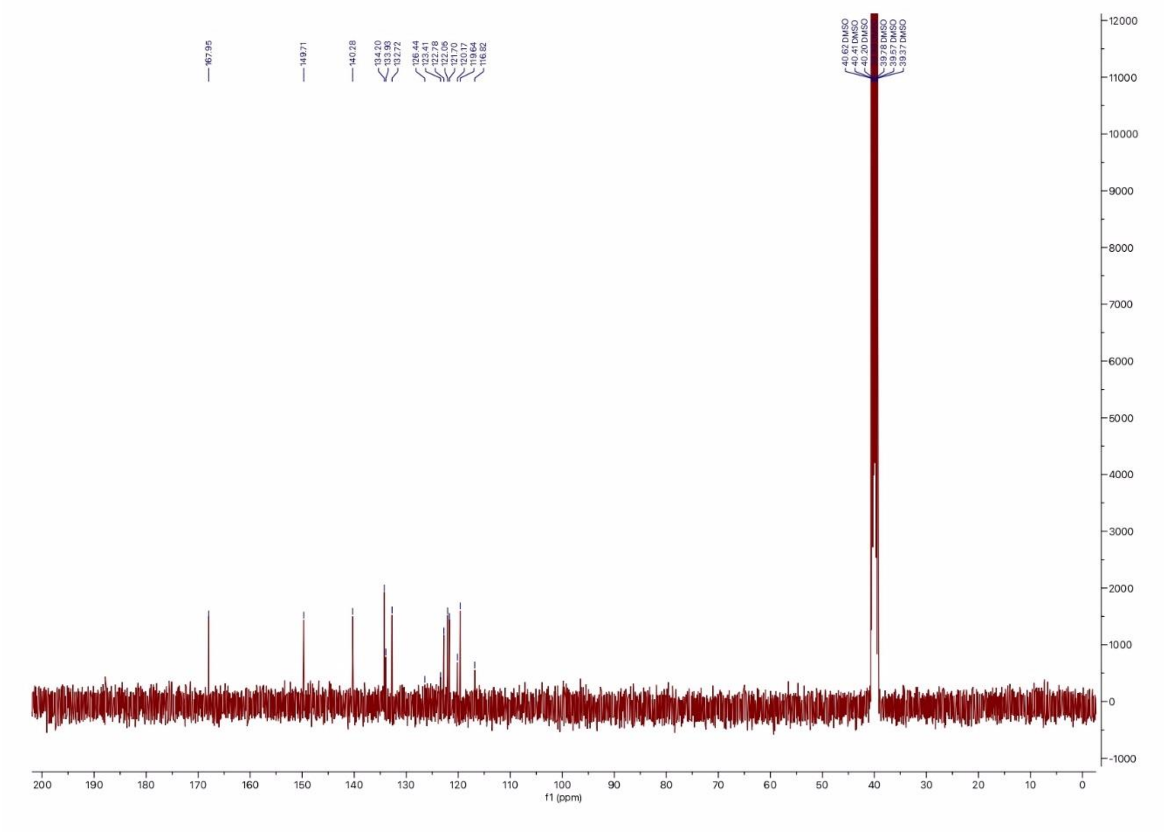

V.7– NMR data for compound 4g

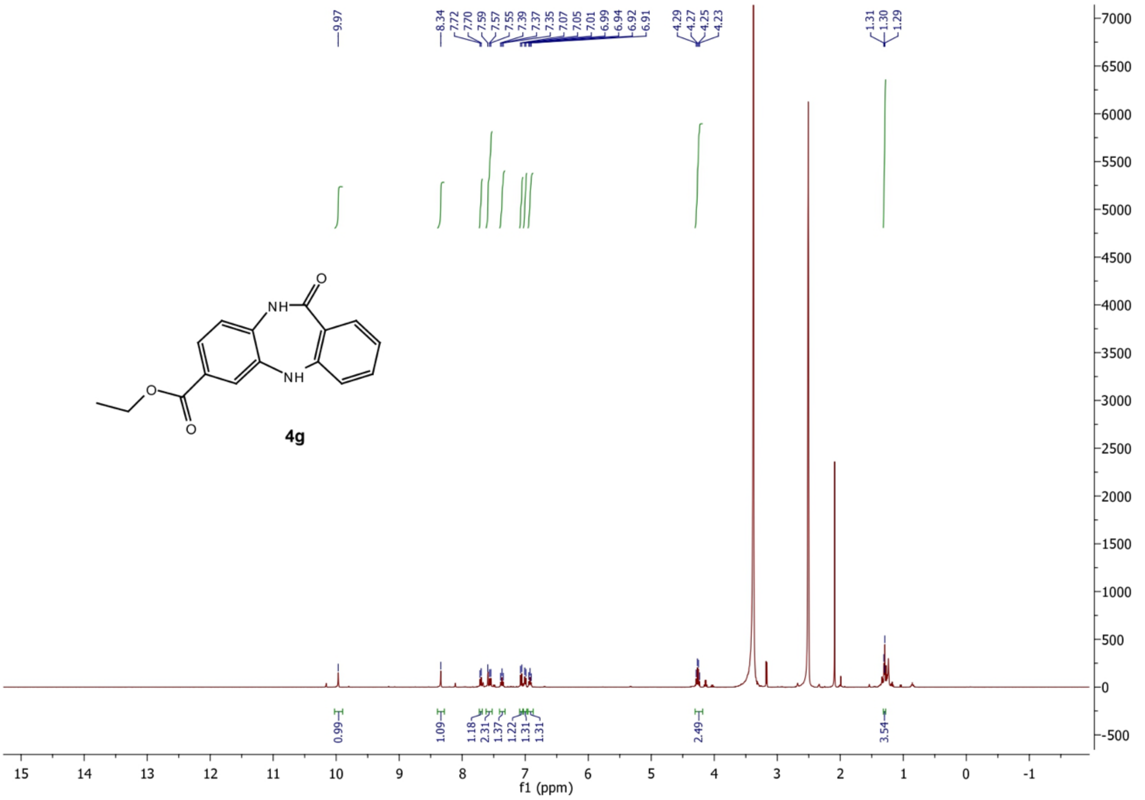

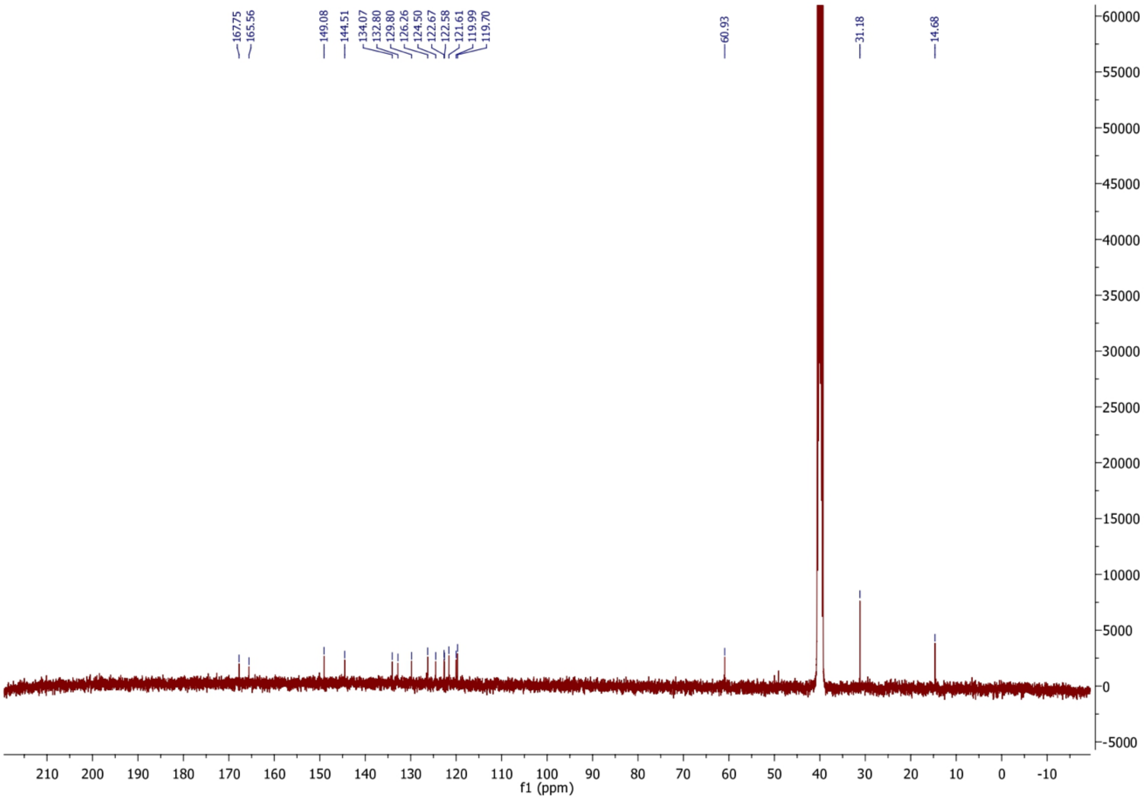

V.8 – Mass spectrum of compound 4h

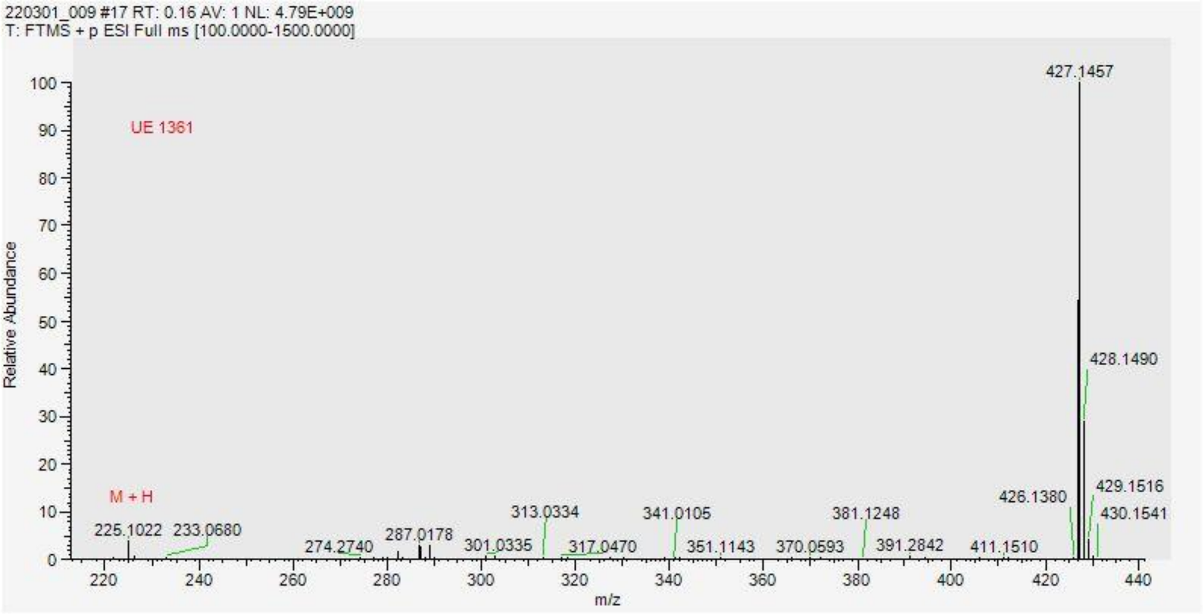

References

1. Tryniszewski, M.; Bujok, R.; Gańczarczyk, R. Wróbel, Z. *Synthesis* **2020**, 52, 3086-3094.
2. Majumdar, K.C., Ganai, S. *Synlett*, **2011**, 13, 1881-1887.
3. Jamatia, R., Gupta, A., Pal, A.K. *ChemistrySelect*, **2016**, 1, 852-860.
4. Iwasawa, T., Tokunaga, M., Obora, Y., Tsuji, Y. *J. Am. Chem. Soc.* **2004**, 126, 6554-6555.
